# A systematic review and meta analysis of open label placebo effects in chronic musculoskeletal pain

**DOI:** 10.1038/s41598-025-09415-y

**Published:** 2025-07-05

**Authors:** Fredrik Borg, Filip Gedin, Erika Franzén, Wilhelmus Johannes Andreas Grooten

**Affiliations:** 1 Division of Physiotherapy, Department of Neurobiology, Care Sciences and Society, Karolinska Institutet, Alfred Nobels Allé 23, Huddinge, 141 83 Stockholm, Sweden; 2 Scandinavian College of Chiropractic, Campus Solna, Fogdevreten 2A, Solna, 171 65 Stockholm, Sweden; 3 Department of Clinical Neuroscience, Karolinska Institutet, Stockholm, Sweden; 4 Medical Unit Allied Health Professionals, Karolinska University Hospital, Stockholm, Sweden

**Keywords:** Long-lasting pain, Nonspecific pain, Back pain, Knee pain, Placebo, Literature review, Chronic pain, Placebo effect, Pain management

## Abstract

Placebo effects have been linked to a range of clinical symptoms. Traditionally, it was believed that deception was necessary for placebos to work. However, emerging research shows that openly administered placebos can benefit patients with various medical conditions. This study aimed to analyze the effects of Open-Label Placebo (OLP) on physical function and pain in individuals with chronic musculoskeletal pain. We conducted, in August 2024, a systematic literature search in PubMed, Web of Science, PsycINFO, and EMBASE for RCTs comparing OLP with any other intervention or no treatment. The level of evidence was assessed with GRADE. The effects on physical function tests were analyzed descriptively, while patient-reported outcome measures (PROMs) on physical functioning and pain were synthesized using random-effects meta-analyses. Study quality was assessed using the revised Cochrane risk of bias tool, RoB2. From 1775 articles, seven RCTs with a total of 703 participants were included—five on back pain and two on knee pain. All seven studies had a high risk of bias for PROMs due to awareness of the intervention. Among the four studies that reported objective outcomes, two had a low risk of bias and two had some concerns. All studies included a follow-up between 3 and 12 weeks, except for one study that had a 3-year follow-up. With moderate evidence, we found that OLP as a stand-alone or add-on treatment had no effect on physical function tests, but concerning PROMs, there was low evidence for very small to moderate effects on self-reported physical function (SMD = 0.40, 95% CI 0.19–0.60) and small to moderate effects on pain intensity (SMD = 0.46, 95% CI 0.28–0.63) compared to treatment as usual or no treatment. These results suggest that OLP may enhance PROMs in chronic musculoskeletal pain patients, but one should keep in mind that the clinical value of OLP is not yet established. This study received no specific funding.

Registered in PROSPERO: CRD42023487578.

**Supplementary Information:**

The online version contains supplementary material available at 10.1038/s41598-025-09415-y.

## Introduction

Chronic musculoskeletal pain (CMP) is a leading cause of disability worldwide^1^, with low back pain being the most prevalent condition^2,3^. From 1990 to 2015, the years lived with disability due to low back pain increased by 54%, primarily due to population growth and aging^4,5^. There is therefore an urgent need for effective and affordable strategies^1^. To address CMP effectively, a holistic, multidisciplinary approach^6,7^, that incorporates various strategies to meet the diverse needs of patients may be required^8^. Despite these efforts, healthcare providers face significant challenges due to the complex nature of CMP. Moreover, one of the most pressing issues is the widespread use of opioids, which has led to significant problems, including addiction and increased mortality rates^9^. This underscores the critical necessity for non-pharmacological pain management strategies, among which chiropractic and physical therapy have been found to be effective in the treatment of musculoskeletal problems^10–12^. However, the level of evidence seldom reaches the highest level of certainty. One reason for this could be that the quality of RCTs in these fields is often ranked as “low” since blinding of patients and therapists is not just challenging but virtually impossible. One way of increasing the quality of these RCTs is to include placebo treatment, where especially Open-Label Placebo (OLP) as an add-on treatment could play an important role. OLP has emerged as an innovative approach to pain management, including CMP, aiming to harness the placebo effects observed in research and clinical settings^13^. Unlike traditional placebos, OLP involves the administration of inactive substances with full transparency, allowing patients to know that they are receiving a placebo^14^. Most OLP studies use pills composed of inert substances, such as microcrystalline cellulose, or saline injections, which have no known effects on pathophysiology^15^. This approach challenges the conventional belief that placebos require either deception or concealment to be effective^16^. Compared to a hidden placebo, this approach better aligns with ethical principles of transparency, respect, and informed consent^17^. In OLP studies, researchers avoid making promises like ‘it will work,’ adopting instead a stance of ‘let’s see what happens’, and if patients express skepticism, researchers openly share their own uncertainties to provide support, fostering a transparent and supportive environment^18^. Typically, these studies involve explaining the powerful placebo effect, comparing the body’s response to placebo pills with Pavlov’s dogs salivating, and acknowledging that while a positive attitude can be helpful, it is not necessary. However, the effectiveness of OLP may be influenced by how these elements are presented and explained, though this impact is not yet fully understood^19^.

One proposed mechanism is *cognitive dissonance*. This occurs when someone takes a placebo while knowing it is inactive yet still experiencing relief. Unconsciously, they might adjust their expectations to resolve the contradiction, but predictive processing models, including prediction and error processing and the Bayesian brain framework, could also play a role in this respect^16,20,21^. These models suggest that the placebo effects may arise when the brain while being “in conflict with itself”, changes the interpretation of incoming sensory information, and thereby influences symptom perception. Classical conditioning, once considered the primary explanation, may instead be part of this broader predictive system. Whereas past experiences and activities – what you previously have been doing and positively resolved the problem – shape responses to treatment. Citing Kaptchuck^20^: “Placebo effects are primarily elicited by what you do, and only secondarily— or not at all — by what you think”. Together, these mechanisms suggest that OLP influences how the brain processes symptoms, enhancing the body’s natural capacity for symptom relief.

Previous research has demonstrated promising results that OLP may improve outcomes for a range of conditions and symptoms, including back pain^22^, IBS^13^, ADHD^23^, allergic rhinitis^24^, cancer-related fatigue^25^, menopausal hot flashes^26^, and migraine^27^, as well as in experimental studies with non-clinical samples^28–32^. However, despite its potential, introducing OLP into clinical settings may be challenging, and its clinical utility remains debated. For chronic musculoskeletal problems, there is moderate evidence that several treatment therapies are effective for functional disability measured clinically or using patient ratings. Consequently, it is argued that OLP should not be used as a stand-alone treatment or as an add-on treatment until more is known about its effectiveness^33^. Moreover, concerns have been raised about the acceptability among patients, especially those with CMP, and the risk of unintended harms, such as external and internal stigma, shame, or delays in proper treatment and diagnosis^34^. On the other hand, several studies show that physicians already prescribe deceptive placebos regularly^35,36^, and there are indications that patients might find placebo treatments acceptable in some circumstances^37^. Nonetheless, further research is needed to clarify the role of OLP and the specific conditions under which it may be effective^38^.

Given that the most recent systematic review by von Wernsdorff et al.^39^ identified only two studies on CMP within the field of OLP, and considering the emergence of several new publications since then, we are conducting a new systematic review. Moreover, to our knowledge, no systematic review has yet assessed the effects of OLP on both physical function tests and self-reported physical function and pain intensity in individuals with CMP. This study aims to fill this gap by systematically reviewing and analyzing the effect of OLP on these outcomes in CMP patients.

## Methods

This systematic review and meta-analysis was reported according to the Preferred Reporting Items for Systematic Reviews and Meta-Analyses (PRISMA) reporting guidelines^40^ (see Supplementary Online Content S1). A protocol was registered a priori with PROSPERO, Centre for Reviews and Dissemination, University of York, CRD-register (CRD42023487578).

Three authors (FB, FG, and WG) worked independently in pairs through all different stages of the review: selecting articles, assessing the relevance and quality of the selected articles, extracting data, and grading the certainty of the evidence of the effects. One junior reviewer (FB) assessed all studies, while the articles were divided randomly between 2 other senior researchers (FG and WG). Consensus on final ratings per domain was reached through discussions within the team and the fourth senior author (EF) was included in the process when conflicts could not be solved.

### Data sources and search procedure

An electronic search was conducted in September 2023 and updated on August 28, 2024, across four databases: PubMed, Web of Science, PsycINFO, and EMBASE (Supplementary Online Content S2). Before conducting the search, two primary concepts central to the research question were defined: chronic musculoskeletal pain (the problem) and open-label placebo (the intervention). Keywords, including MeSH terms, free-text terms, and specific phrases, were then identified and extracted from previous studies^16,39,41,42^, and a MeSH database^43^. Following consultation with the Karolinska Institute library, we developed search strategies around these concepts and combined them using Boolean operators. Various filters were applied in different databases, including those for human participants and randomized controlled trials (RCTs) in EMBASE, and a filter for human participants in PsycINFO. No filters were used in the other databases. Only RCTs were considered for this review. To broaden the search, terms related to specific outcomes were intentionally omitted. Additionally, searches were conducted within the reference lists of included studies to ensure comprehensive coverage.

### Study selection

The search results from each respective database were imported to Zotero^44^ for identification and removal of duplicates. Following this, the remaining articles were uploaded to Rayyan^45^ for screening of titles and abstracts against inclusion and exclusion criteria, which were based on the acronym PICOS (see Table 1).

**Table 1:** Inclusion and exclusion criteria.

PICOS	Inclusion	Exclusion
Population	Adults with chronic musculoskeletal pain	healthy individuals, cancer pain, fractures, infections, rheumatoid arthritis, fibromyalgia, neurological disorders
Intervention	Open label placebo or add-on open label placebo	
Control	No restriction to the control group	
Outcome	Primary outcomes are physical measures of movement performance during daily activities, such as bending capacity (e.g. Schober test/fingertips to floor test), raising from a chair, lifting capacity or gait, and other objective measures such as range of motion and muscle strength. Secondary outcomes include subjective measures of self-administered functioning and pain scales (e.g. ODI, NPRS)	Psychological and behavioral health assessment, measures of quality of life, economic evaluations, fMRI
Study design	RCT	
Additional aspects	Papers in English, Swedish, Norwegian, Dutch, German	

RCT, Randomized controlled trial; fMRI, functional magnetic resonance imaging; ODI, Oswestry disability index; NPRS, Numeric pain rating scale.

### Data extraction

The following information from each included article was extracted: (i) trial characteristics: title, author’s name, publication year, country, and aim, (ii) demographics: quantity of participants, age, sex, pain site, and pain duration, (iii) intervention: placebo and intervention type(s), participant instruction and procedure, dose and the number of sessions, and eventual co-intervention(s), (iv) outcomes: tests of physical functioning of movement performance, and patient-reported outcome measures (PROMs) of physical function and pain-intensity, (v) results: participation rate, eventual adverse events, all results from tests of physical functioning and PROMs of physical functioning and pain intensity. For all studies, data at short-term follow-up (3–12 weeks) was extracted, except for Kleine-Borgmann et al.^46^, in which a 3-year follow-up was reported. In all studies data on mean between-group differences was provided, but in Ashar et al.^47^, and Bandak et al.^48^, the mean between-group differences were calculated from the data presented.

### Quality assessment/risk of bias

The included articles were assessed for risk of bias (RoB) using the RoB 2 tool (the revised Cochrane risk of bias tool for randomized trials)^49^. The potential threats to validity were assessed using intention-to-treat (ITT) analysis within five domains for each outcome and time points: (i) bias arising from the randomization process, (ii) bias due to deviations from intended interventions, (iii) bias due to missing outcome data, (iv) bias in the measurement of the outcome, (v) bias in the selection of the reported result. If agreement couldn’t be achieved, a third author was responsible for making the ultimate decision. The process for assessing RoB was evaluated for interrater agreement, with percent agreement calculated using a linear weighted kappa (*k*)^50^.

### Data analyses

RevMan 5.4 was used as statistical software^51^. Effect sizes were assessed using Hedges’ g, which compares post-treatment means between groups, resulting in Standardized Mean Differences (SMDs) for the treatments based on standard deviations (SDs). A meta-synthesis of the effect sizes was performed using a random-effects model. Effects were classified as very small positive effect size when the SMD was between 0 and − 0.2, small positive effect size when between − 0.2 and − 0.5, moderate positive effect size when between − 0.5 and − 0.8, and large positive effect size when less than − 0.8^52^. *I²* statistics were calculated to judge the heterogeneity and a value larger than 75% was interpreted as considerable heterogeneity^53^. To assess the risk of publication bias, we checked for unpublished data by searching in the trials register (https://clinicaltrials.gov/), protocols or statistical analysis plans (SAPs) and results published in conference abstracts or the grey literature. Moreover, the SAPS of the included studies were studied in order to check for selected reporting. Sensitivity analyses were conducted for pain sites, including back pain and knee pain. Additionally, we tested correlations between year of publication, and study size against study effect size.

### Grade

The level of evidence (certainty of evidence) for each outcome was assessed in four levels (high, moderate, low and very low) using the GRADE approach^54^, and GRADEpro GDT software^55^. In short, the quality of evidence is determined by five factors that can reduce the certainty (limitations in study design, inconsistency in results, indirectness of evidence, imprecision, and publication bias) and three factors that can increase the certainty (magnitudes of effects, plausible confounding, and dose-response gradient). The highest level of evidence was chosen as the starting level, since only RCTs were included.

## Results

### Study selection

**Fig. 1:**
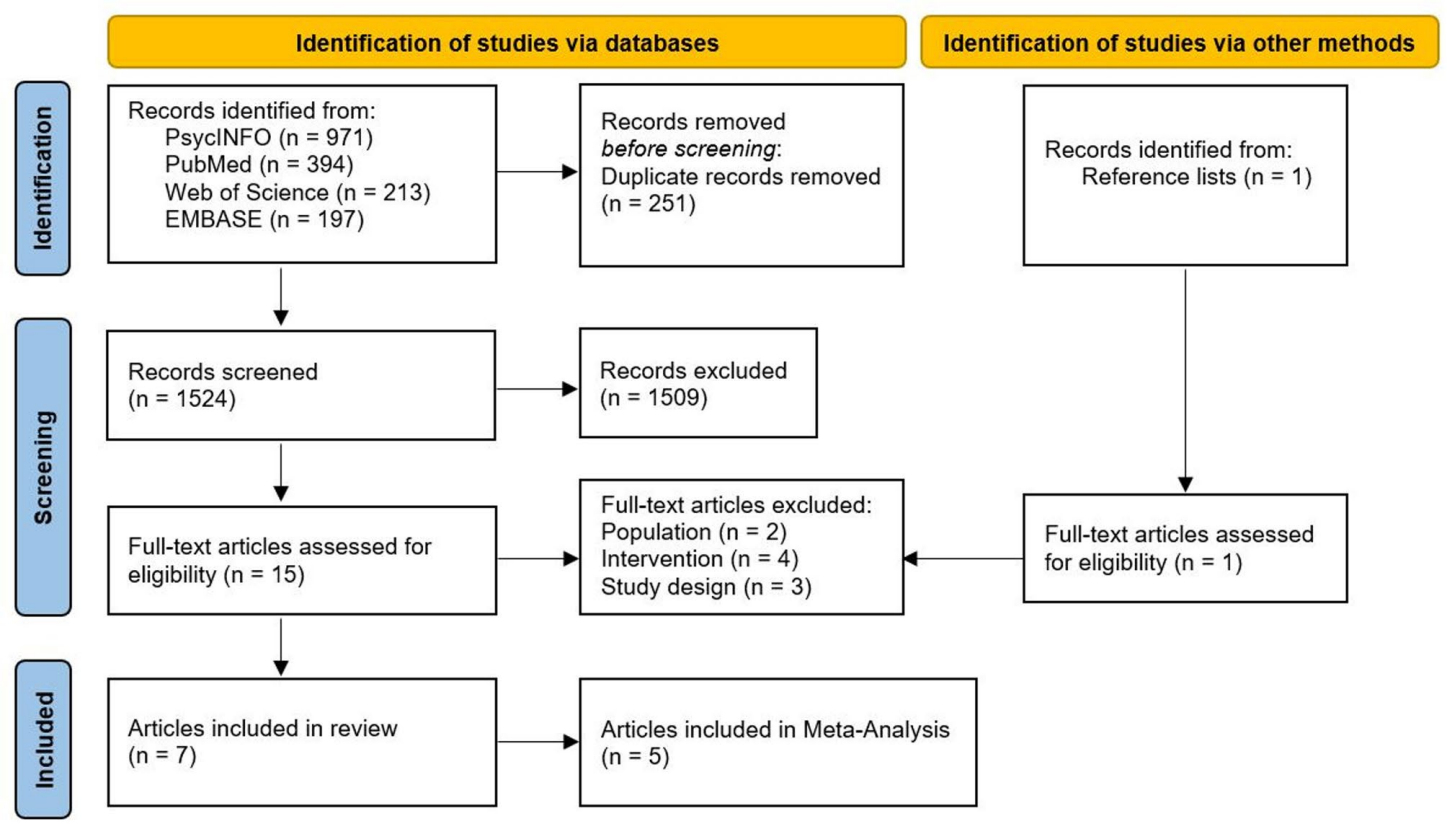
PRISMA flow chart.

The search yielded 1775 articles, with 251 duplicates. After screening 1524 articles and reviewing 16 in full text, seven met the inclusion criteria—five involving individuals with back pain (*n* = 437)^22,46,47,56,57^, and two with knee pain (*n* = 266)^48,58^. All articles included PROMs of physical function and pain, with four studies also including tests of physical function^22,48,56,57^. Due to fundamental differences in the outcomes in the four studies that studied tests of physical function, no meta-analysis was performed, and the results were analyzed descriptively. Five of the seven studies were qualified for meta-analysis for PROMs of both physical function and pain. Neither the study by Bandak et al.^48^, which employed an active comparator, nor the study by Kleine-Borgmann et al.^46^, which reported long-term outcomes at a three-year follow-up, were included in the meta-analysis. The latter included the same subjects as the primary study by Kleine Borgmann et al.^57^, violating the assumption of independence of effect sizes. Both studies were retained for descriptive analysis. For specific details of the screening process and numbers, please refer to Fig. 1. The reasons for excluding the nine studies that were read in full text (Supplementary Online Content S3).

### Characteristics of studies

A total of 703 participants were included in the studies. Two studies focused on individuals with knee osteoarthritis^48,58^, while five studies addressed chronic back pain^22,46,47,56,57^. The trials were conducted in Europe (Germany, Denmark, Portugal), North America (USA), and Asia (Japan). For the control group, “treatment as usual” (TAU) was employed in five studies, with variations across different studies. Additionally, one study used “exercise and education” as the control, and one study employed “no treatment” (NT) as the control (see Table 2).

**Table 2:** Study characteristics - Description of population, intervention, and control group.

Authors, year, country	Population			Intervention			Control
	Diagnosis, number of subjects, ethnicity	Age mean (SD), % female	Duration of pain: mean (SD)	Type, dose	Participant instruction	Monitoring, co-intervention, adverse events (AE)	Type
Carvalho et al.^22^, 2016, Portugal	CLBP, *n* = 83, N.R.	44.3 (13.5), 71	≥ 3 month	The placebo pills were Swedish Orange gelatin capsules filled with microcrystalline cellulose, 42 capsules in 21 days	The PI explained that the placebo pill was an inactive substance, similar to a flour pill. Participants were briefed on the placebo effect using a 15-minute explanation covering: (1) its power, (2) automatic bodily responses, (3) the role of a positive attitude, and (4) the importance of consistent use for 21 days. They also watched a 1-minute, 25-second news clip about an OLP trial for irritable bowel syndrome.	Unmonitored. Co-interventions: Pain medication use: OLP 85.4% and Control 88.1%. AE: Almost non-existent	TAU: not defined
Kleine-Borgmann et al.^57^, 2019, Germany	CLBP, *n* = 127 total, 122 analysed, N.R	59.2 (14.6), 63	≥ 3 month	Placebo capsules containing microcrystalline cellulose (Zeebo Effect, LLC, South Burlington, Vermont), 42 capsules in 21 days	All patients were informed that the capsules did not contain any active ingredients. They also watched a 1-minute, 25-second news clip about an OLP trial for irritable bowel syndrome	Unmonitored. Co-interventions: Pain medication use: OLP 22% and Control 20% AE: None	TAU: received no intervention. TAU was defined as any pharmacological or nonpharmacological treatment. All TAU group patients were offered the same OLP treatment upon completion of the study
Ikemoto et al.^56^, 2020, Japan	CLBP, *n* = 52, Japanese	66.8(13.4), 68	6 month-1 year: 23.1% 1–5 years: 50.0% > 5 years: 26.9%	The placebo pills were gelatin capsules filled with Lactose (450 mg per capsule), 168 capsules in 12 weeks	Patients were informed that the placebo capsules did not contain any active ingredients. Patients were provided with standardized information on the placebo effect. Briefly, this information covered “five points”: (1) the placebo effect is powerful; (2) the placebo effect has been confirmed in the previous literature; (3) the body can automatically respond to a placebo capsule like Pavlov’s dogs which salivated when they heard a bell; (4) a positive attitude helps but is not necessary; and (5) taking the pills faithfully is critical	Unmonitored. Co-interventions: Pain medication use: OLP 61.5% and Control 65.4%. AE: N.R	TAU included advice to remain active along with education and reassurance as first-line care. Moreover, a recently described psychological education based on a self-management strategy was used to improve pain-related disabilities
Ashar et al.^47^, 2021, USA	CBP, *n* = 151 total, 88 analysed, American Indian or Alaskan Native: OLP: 0%; Control: 2%; Asian/Pacific Islander: OLP: 4%; Control: 0%; Black (not of Hispanic origin): OLP: 4%; Control: 2%; White (not of Hispanic origin): OLP: 88%; Control: 86%; Other or unknown: OLP: 4%; Control: 10%; Hispanic ethnicity: OLP: 4%; Control: 4%	41.1 (15.6), 54	10.0 (8.9)	Subcutaneous injection described as saline at the site of greatest back pain, 1 injection in 4 weeks	Participants watched 2 videos describing how placebo treatments can powerfully relieve pain even when known to be inert (e.g. it can automatically trigger the body’s natural healing response)	Monitored. Co-interventions: Opioid use: OLP group 4% and Usual Care 4%. AE: N.R	TAU: No new treatments before the post-treatment assessment. Not further described
Bandak et al.^48^, 2022, Denmark	Knee OA, *n* = 206, N.R	68.4 (8.5), 46	N.R	Intra-articular saline injections, 4 injections distributed over weeks 1, 3, 5, and 7 after baseline	Information was delivered neutrally, ensuring that descriptions of both interventions were promoted equally including that the investigators had no treatment preference. Saline injections were described as inert, yet with potential beneficial effects that may compare to those of exercise and education. The participants were informed that ‘active ingredients’ in both interventions are unverified and involve the sum and interaction of many factors	Monitored Co-interventions: Paracetamol/NSAID OLP group 41.4% and Control 34%. AE: similar in the two groups, OLP: 39% and Control: 34% None of the serious AE’s appeared related to the study treatment	Exercise and Education: Two group education sessions and supervised neuromuscular exercise with body weight and/or resistance band(s)
Olliges et al.^58^, 2022, Germany	Knee OA, *n* = 60, N.R	66.9 (9.5), 55	≥ 6 month	Placebo capsules filled with mannitol, 42 capsules in 21 days	Participants received general information on the placebo effect, namely that the placebo effect is powerful, the body can automatically respondtotaking placebo pills like Pavlov’s dogs who salivated when they heard a bell, a positive attitude helps but is not necessary, and taking the pills faithfully is critical. Also, either instruction: “to relieve pain” or to “improve mood”	Unmonitored. Co-interventions: None. AE: N.R	No treatment. The control group received no intervention
Kleine-Borgmann et al.^46^, 2023, Germany	CLBP, *n* = 89, N.R	60.5 (2.7), 69	≥ 3 month	Placebo capsules containing microcrystalline cellulose (Zeebo Effect, LLC, South Burlington, Vermont), 42 capsules in 21 days	All patients were informed that the capsules did not contain any active ingredients. They also watched a 1-minute, 25-second news clip about an OLP trial for irritable bowel syndrome	Unmonitored. Co-interventions: Groups did not differ in any other exploratory outcomes incl. pain medication. AE: N.R.	TAU: received no intervention. TAU was defined as any pharmacological or nonpharmacological treatment. All TAU group patients were offered the same OLP treatment upon completion of the study

OLP, Open-label placebo; CLBP, Chronic low back pain; CBP, Chronic back pain; OA, osteoarthritis; N.R., Not reported; SD, Standard deviation; AE, Adverse events; NSAID, Non-steroidal anti-inflammatory drugs; TAU, Treatment as usual.

### Characteristics of participants

The mean age ranged from 41.1 (SD 15.6)^47^ to 68.4 (SD 8.3)^48^ years. The mean duration of pain varied from ≥ 3 months to 10 (SD 8.9) years. The proportion of women in six of the studies ranged from 54 to 71%^22,46,47,56–58^, while one study reported that 54% of participants were men.

### Treatment format

In five studies^22,46,56–58^, the OLP consisted of capsules containing microcrystalline cellulose, lactose, or mannitol, taken unmonitored twice daily for either 3 or 12 weeks. In two studies^47,48^, the OLP involved saline injections, administered either once in 4 weeks or four times over 6 weeks.

### Outcome reporting and assumptions required for synthesis

**Fig. 2:**
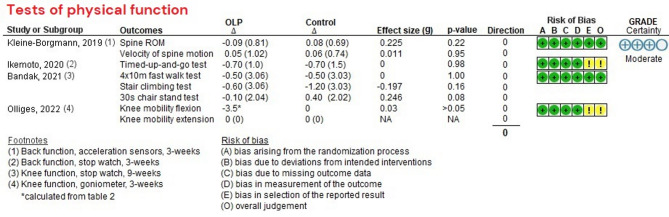
Descriptive analysis for tests of physical function. OLP∆, Change in OLP group; Control∆, Change in control group; g, Hedge’s g; Direction 0, indicates no effect favoring either OLP or the control group.

**Fig. 3:**
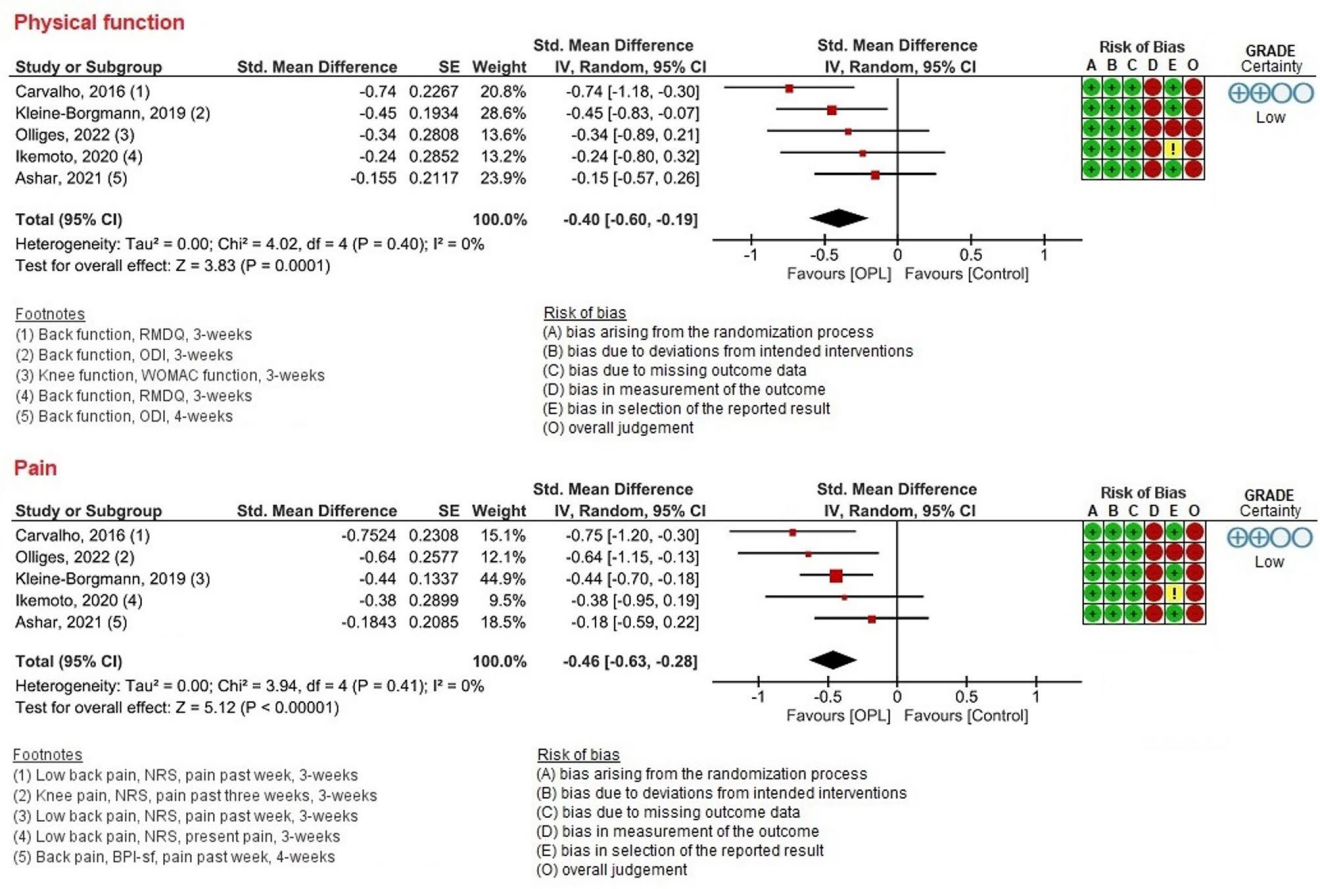
Forest plots for PROMs of physical function and pain.

Outcomes were measured as follows: Tests of physical function included clinical tests, including the Timed-Up-and-Go test (TUG), 4 × 10 m fast walk test, Stair Climbing test, and 30s Chair Stand test, as well as range of motion (ROM), and velocity of spine motion (see Fig. 2). Concerning the PROMs of physical function, KOOS and WOMAC were used in studies on chronic knee pain, and ODI and RMDQ were used in studies on chronic back pain (see Fig. 3), while patients reported their pain intensity with NRS or VAS (see Fig. 3). All studies reported outcomes post-intervention (3–12 weeks), except for one study with a 3-year follow-up^46^. Additionally, one study also included measurements at additional time points of 6, and 12 months^47^. However, only post-intervention data from 3- and 4-week time points (i.e., short-term) were included in the meta-analyses, in order to decrease heterogeneity between the studies.

### Individual study results

Carvalho et al.^22^ investigated the effects of OLP as an add-on to TAU in persons with chronic low back pain. Forty-two persons received only TAU, and 41 received OLP—two capsules per day—along with TAU for 21 days. Pain intensity and back-related disability were measured using NRS and RMDQ. After 3 weeks, the OLP group showed significant improvements in both pain (*p* < 0.001) and disability (*p* < 0.001).

Kleine-Borgmann et al.^57^ studied the effects of OLP on chronic back pain. One group (*n* = 63) received OLP—two capsules per day—along with TAU (OLP + TAU) for 21 days, while the other group (*n* = 59) received TAU alone. Physical function and pain were measured using ODI and NRS. ROM and spine motion velocity were evaluated using acceleration sensors. ODI and NRS scores improved significantly in the OLP + TAU group (*p* = 0.001) compared to TAU. No significant differences were observed between groups in the physical function tests.

Ikemoto et al.^56^ examined the effects of OLP as an add-on to TAU in persons with chronic pain. Twenty-six persons received only TAU, while 26 received OLP—two capsules per day—alongside TAU for 21 days. Pain intensity and back-related disability were measured using the NRS and RMDQ, while physical function was evaluated with the TUG. No significant differences were found between groups at the 3-week follow-up: RMDQ (*p* = 0.40), NRS (*p* = 0.19), and TUG (*p* = 0.98), these findings persisted at the 12-week follow-up.

Ashar et al.^47^ conducted a 4-week RCT investigating the effects of OLP administered via subcutaneous injection in individuals with long-term back pain. Fifty-one participants received OLP, which consisted of watching two informational videos followed by a single subcutaneous injection (saline, described openly as placebo) at the site of greatest back pain; 50 participants received TAU alone. Pain intensity and disability were measured using NRS and ODI. At post-intervention, no significant differences were observed between the OLP and TAU groups for either outcome: NRS (p = n.s.) and ODI (p = n.s.).

Bandak et al.^48^ investigated the effects of OLP in an 8-week RCT on individuals with knee osteoarthritis. One group (*n* = 104) received OLP as intra-articular saline injections four times over 8 weeks, while the other group (*n* = 102) received exercise and education. The saline injections were described as inert, yet with potential beneficial effects that may compare to those of exercise and education. Participants were informed that the ‘active ingredients’ in both interventions were unverified and likely involved the sum and interaction of multiple factors. The exercise and education group attended group-based sessions twice a week for six weeks, with each session lasting one hour. Tests of physical function included the 4 × 10 m fast walk test, Chair Stand Test, and Stair Ascend and Descend Test. Pain intensity and disability were measured using VAS and KOOS. The results showed that OLP was as effective as the 8-week exercise and education program in improving symptoms and physical function in individuals with knee osteoarthritis.

Olliges et al.^58^ conducted a 3-week RCT where 60 persons were randomized to receive one of two OLP treatments (*n* = 41) or no treatment (NT; *n* = 19). The OLP groups received verbal instructions “to decrease pain” (*n* = 20) or “to improve mood” (*n* = 21) and took two OLP capsules per day. Pain intensity (NRS), symptoms and function (WOMAC), and knee mobility were measured. Results showed a significant reduction in knee pain in the OLP groups compared to NT (*p* = 0.013) and significant improvement in WOMAC pain scores (*p* = 0.036), with no differences between the two OLP groups. Other measures did not differ significantly between groups.

Kleine-Borgmann et al.^46^ conducted a 3-year follow-up of their primary study, Kleine-Borgmann et al.^57^, analyzing data from 89 participants (47 in the OLP + TAU group and 42 in the TAU group). Pain intensity was assessed using NRS, and disability with ODI. This long-term follow-up showed no significant differences between groups in pain intensity (SMD = 0.05, *p* = 0.685) or disability (SMD = 0.02, *p* = 0.888).

### Risk of bias

The RoB in studies on physical tests was low in two studies while two had some concerns (see Fig. 2). For PROMs of physical function and pain intensity, all studies were rated as having a high risk of bias due to awareness of the intervention, which represent a high risk under Domain 4 of the RoB 2 (see Fig. 3). The interrater agreement for the RoB assessment showed strong agreement between the junior and senior raters; *k* = 0.832 (95% CI 0.75–0.91) for subjective outcomes and *k* = 0.877 (95% CI 0.80–0.95) for measured outcomes.

### Tests of physical function

We analyzed the results descriptively since a meta-analysis was not feasible. Four studies included different tests of physical function as their outcomes and the descriptive analyses showed that none of the studies showed any significant effects, compared to any of the control groups: TAU, NT, exercise & education as add-on or stand-alone interventions (see Fig. 2). Our GRADE analysis showed a moderate level of certainty for no effects of OLP on tests of physical function compared to any other treatment. We downgraded due to imprecision due to large 95%CI’s in all studies. For details of the GRADE assessments (supplementary online content S4).

### PROMs

We found a significant small effect size of OLP as add-on intervention / stand-alone intervention for self-reported physical function (SMD = 0.40, 95% CI 0.19–0.60, *p =* 0.0001, *I²* = 0%) and a small effect size for pain intensity (SMD = 0.46, 95% CI 0.28–0.63, p = < 0.00001, *I*^2^ = 0%) compared to treatment as usual or no treatment (see Fig. 3). GRADE analyses showed a low level of certainty for a small effect of OLP as add-on treatment for PROMs of physical function and pain intensity. We downgraded due to risk of bias and imprecision. For details of the GRADE assessments (supplementary online content S4).

### Potential publication bias

There were no additional OLP studies available in trial registers, protocols, SAPs, and the grey literature, indicating no direct signs of publication bias.

### Sensitivity analyses

**Fig. 4:**
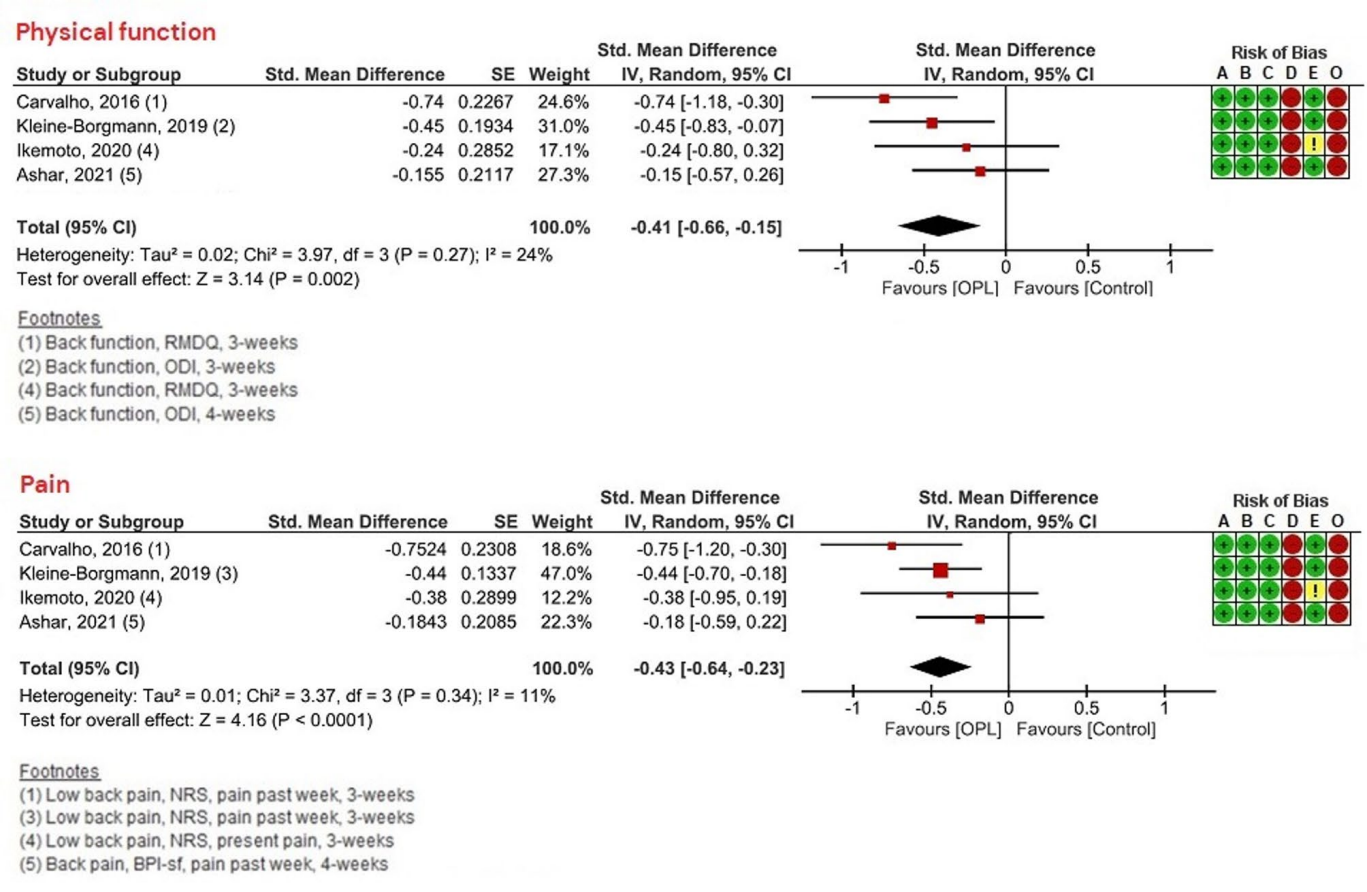
Sensitivity and subgroup analyses—only studies on back pain.

**Fig. 5:**
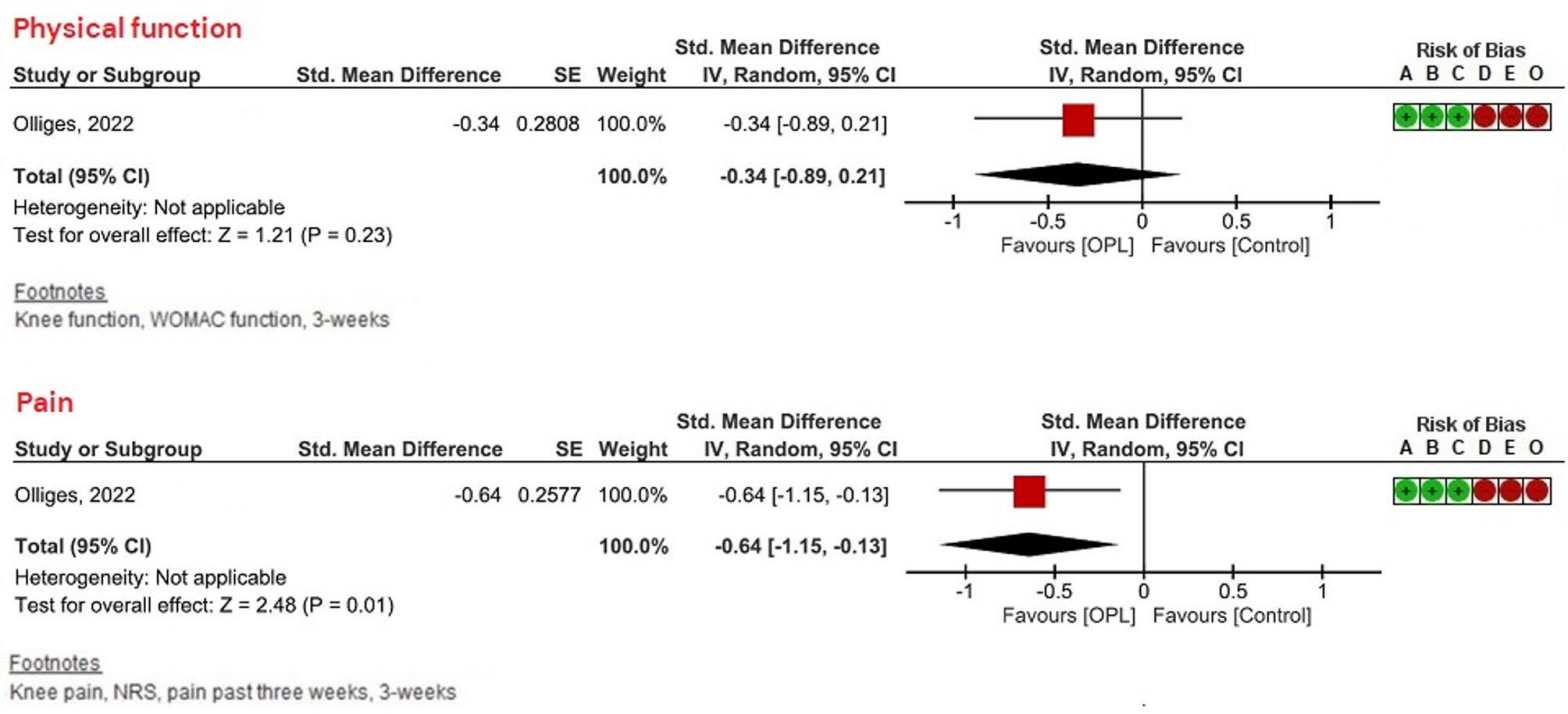
Sensitivity and subgroup analyses—only studies on knee pain.

Our sensitivity analyses showed that OLP, compared to TAU, was effective for individuals with chronic back pain for both PROMs of physical function and pain intensity (see Fig. 4). For chronic knee pain, OLP was effective for pain intensity compared to NT, but not for physical function (see Fig. 5). Given the limited number of included studies (< 10), these sensitivity analyses should be interpreted with caution.

Analyses of the relationship between publication year and effect size showed a strong negative correlation for physical function (Pearson’s *r* = − 0.863, *p* = 0.0594), which approached statistical significance. In contrast, the correlation for pain intensity was weaker and not statistically significant (Pearson’s *r* = − 0.500, *p* = 0.391). Additionally, no significant association was found between effect size and sample size for either physical function (Pearson’s *r* = 0.277, *p* = 0.652) or pain intensity (Pearson’s *r* = − 0.148, *p* = 0.812) (see Supplementary Online Content S5–S8).

## Discussion

### Summary of findings

The results show, with moderate-certainty evidence, that individuals with CMP do not benefit from OLP if evaluated with tests of physical function. However, OLP seems to have small effects post-intervention on PROMs of physical function and pain intensity in this population, although the certainty of evidence was somewhat lower.

### Interpretation of findings

Although we were not able to pool the results, in our study, none of the eight different clinical tests showed statistically and clinically significant results which makes us conclude that our findings align with previous systematic reviews on OLP, which indicate no effect on tests of physical function^41^, but significant effects on patient-reported outcomes^39,41,42^. Despite differences between our study and the earlier reviews—such as variations in conditions, sample characteristics, and outcomes—our results support the conclusions of these reviews.

While we can assert with moderate-certainty that OLP does not provide additional benefits for persons with chronic musculoskeletal pain when assessed with tests of physical function, the small positive effects observed for patient-related outcomes should be interpreted with caution as future research may produce different results. Additionally, these small effects may not translate automatically into meaningful absolute changes on scales such as NRS, NPRS, ODI, or KOOS. When considering the minimal clinically important change (MCIC), the mean point estimate might fall below the threshold required for a clinically meaningful improvement, raising questions about the clinical importance. Still, the upper 95% confidence interval of the effect size for pain indicated moderate effects and since there is a large between-subject variation visible in all studies, reductions in pain and perceived improvements in physical function could have been clinically important for some individuals.

We were unable to conduct a meta-analysis for tests of physical function due to the variability in clinical tests with different units of outcomes (seconds, number of repetitions) employed across the studies. This issue is further complicated by the inherent limitations of these clinical tests, which often face challenges related to interpretation and precision^59^. These limitations likely contributed to many studies being underpowered to detect effects. Consequently, our study underscores the need for further research using consistent and precise measurement methods. Interestingly, all studies, except for Bandak et al. (2022), showed effects in favor of OLP^48^. This difference could depend on the difference in the control group, wherein Bandak et al. (2022) the control group received an active intervention, in contrast to all other studies where the control group remained passive.

While the meta-syntheses showed significant post-intervention between-group effects concerning PROMs of physical functioning and pain intensity, the sensitivity analyses revealed that OLP effects were significant in individuals with chronic back pain for both outcomes. For those with chronic knee pain, the effect on pain intensity remained significant, whereas the effect on physical function was non-significant. This finding could be noteworthy as previous studies^13,25–27^ have shown that OLP is effective in individuals with more diffuse, widespread pain syndromes, while those with knee pain tend to perceive their pain as specific and localized. It is possible that different pain presentations respond differently to OLP. However, since the sensitivity analyses included only a limited number of studies—just one study on chronic knee pain and four on chronic back pain— findings from these analyses should be interpreted with caution, and further research is needed to clarify these results.

Interestingly, a strong negative correlation between publication year and effect size was observed for physical function, which approached statistical significance. This suggests a trend toward smaller reported effects in more recent studies. This may reflect the well-documented tendency for earlier studies to report larger effect sizes, which often diminish as more rigorous research is conducted^60^.

### Strength and limitations

Several limitations should be acknowledged. First, our language restriction to English, Swedish, Norwegian, Dutch, and German articles may have led to the exclusion of relevant studies. Second, although we searched for grey literature—such as trial registries, protocols, and unpublished results—our search could have been more comprehensive. More rigorous methods for identifying grey literature exist, and a broader search might have identified additional studies. However, as our review focused exclusively on RCTs, which are typically published in peer-reviewed journals, the risk of missing high-quality RCTs is likely low. Still, a more extensive grey literature search could have enhanced the completeness of our review.

The included studies varied in methodological quality. All outcomes based on PROMs were assessed as having a high risk of bias, which limits the certainty of these findings. Despite this, the meta-analyses of PROMs showed no statistical heterogeneity (*I*^2^ = 0%), and the 95% confidence intervals were not wide, suggesting consistent and relatively precise results across studies. However, heterogeneity among the studies with tests of physical function as outcome was substantial due to differences in assessment tools, which limited the possibility to conduct meta-analyses for these outcomes and the generalizability of the results.

As fewer than ten studies were included in the sensitivity analysis, it should be viewed as exploratory. Formal sensitivity analyses based on small numbers of studies are not considered robust, and the results may be less reliable. Therefore, conclusions drawn from these analyses should be interpreted with caution. However, most studies were well-matched in terms of the type and dose of OLP, follow-up periods, as well as the information provided to participants, which contributes positively to the robustness of the results. Although a few studies reported conflicts of interest, these conflicts might not have significantly impacted the results. Additionally, the good inter-rater reliability between the included raters is an additional strength of this study.

### Clinical implications and future research

OLP has the potential to offer a valuable, safe, non-pharmacological alternative to pain management strategies. Although OLP cannot cure diseases, it might be used as a “dose-extender” or “partial reinforcement” method. For instance, in the context of drug use for pain, OLP could initially be paired with these medications and then gradually replace them to potentially reduce the overall dosage (i.e. conditioning)^16^. This strategy might, among other benefits, help support efforts to tackle the opioid crisis^61^. Furthermore, future research could investigate how OLP might complement other standard approaches for CMP. This raises the question of whether others besides doctors could prescribe these inert pills, and whether therapists would be willing to do so.

Moreover, the use of OLP in healthcare offers a promising approach for administering placebos transparently and addressing several ethical concerns traditionally associated with placebo use^62^. However, as noted in previous research, ethical challenges may persist, such as the risk of paternalism^63^, and self-stigmatization^64^. Additionally, there may be a risk of medicalizing problems primarily influenced by social or environmental factors.

To advance our understanding of OLP, it is important to further investigate the mechanisms of OLP, and potential long-term effects, and to identify any differences between various populations, such as those experiencing acute versus chronic pain or specific versus non-specific pain. Moreover, it is important to address several methodological challenges in this field of research, such as the type of information provided to participants, the choice of comparison groups, and the quality of the provider’s interaction with participants. It would also be valuable to consider whether OLP might serve as an alternative to traditional blinding in studies where blinding is difficult to achieve, such as in non-pharmacological trials (e.g., physical, psychological, and self-management interventions), where concerns about research quality arise due to methodological challenges related to control interventions (e.g., placebo controls)^65^, and to explore practices that could optimize its use. Investigating these aspects through further research, especially using physical measures and not only PROMs, could provide deeper insights and help refine guidelines for the effective application of OLP.

## Conclusions

Our findings suggest, with moderate evidence, that OLP does not appear to improve clinical test outcomes of physical function. On the other hand, with low certainty evidence, OLP may offer benefits in enhancing self-reported physical function and reducing pain intensity in individuals with chronic musculoskeletal pain. However, the role of OLP in a clinical setting is not yet established, and given the availability of treatments supported by stronger evidence, its use as a stand-alone treatment is difficult to justify and may raise ethical concerns. Consequently, further research is needed to explore OLP’s potential, in particular as an adjunct to traditional pain management approaches, to better understand its place in both research and clinical practice.

### Deviations from protocol

There were no deviations from the study protocol.

## Electronic supplementary material

Below is the link to the electronic supplementary material.

Supplementary Material 1

Supplementary Material 2

Supplementary Material 3

Supplementary Material 4

Supplementary Material 5

Supplementary Material 6

Supplementary Material 7

Supplementary Material 8

Supplementary Material 9

## Data Availability

Data extracted from the articles, used for the analyses, and the analysis codes can be obtained from the corresponding author upon request. There are no restrictions on data access.
