# Supplementary material for: A systematic review and meta analysis of open label placebo effects in chronic musculoskeletal pain
Source: Sci Rep. 2025 Jul 5;15:24007. doi: 10.1038/s41598-025-09415-y (PMC12228692; doi:10.1038/s41598-025-09415-y)

**Supplementary Online Content**

**Supplementary file S1.** Prisma checklist

**Supplementary file S2.** Databases, search strategy and number of articles retrieved from the database searches

**Supplementary file S3.** Excluded Studies and Reasons for Exclusion

 **Supplementary file S4.** GRADE assessments

**Supplementary file S5.** Correlation between publication year and effect size for PROMs of physical function

**Supplementary file S6.** Correlation between publication year and effect size for PROMs of pain intensity

**Supplementary file S7.** Correlation between sample size and effect size for PROMs of physical function

**Supplementary file S8.** Correlation between sample size and effect size for PROMs of pain intensity

**Supplementary file S1. Prisma checklist**


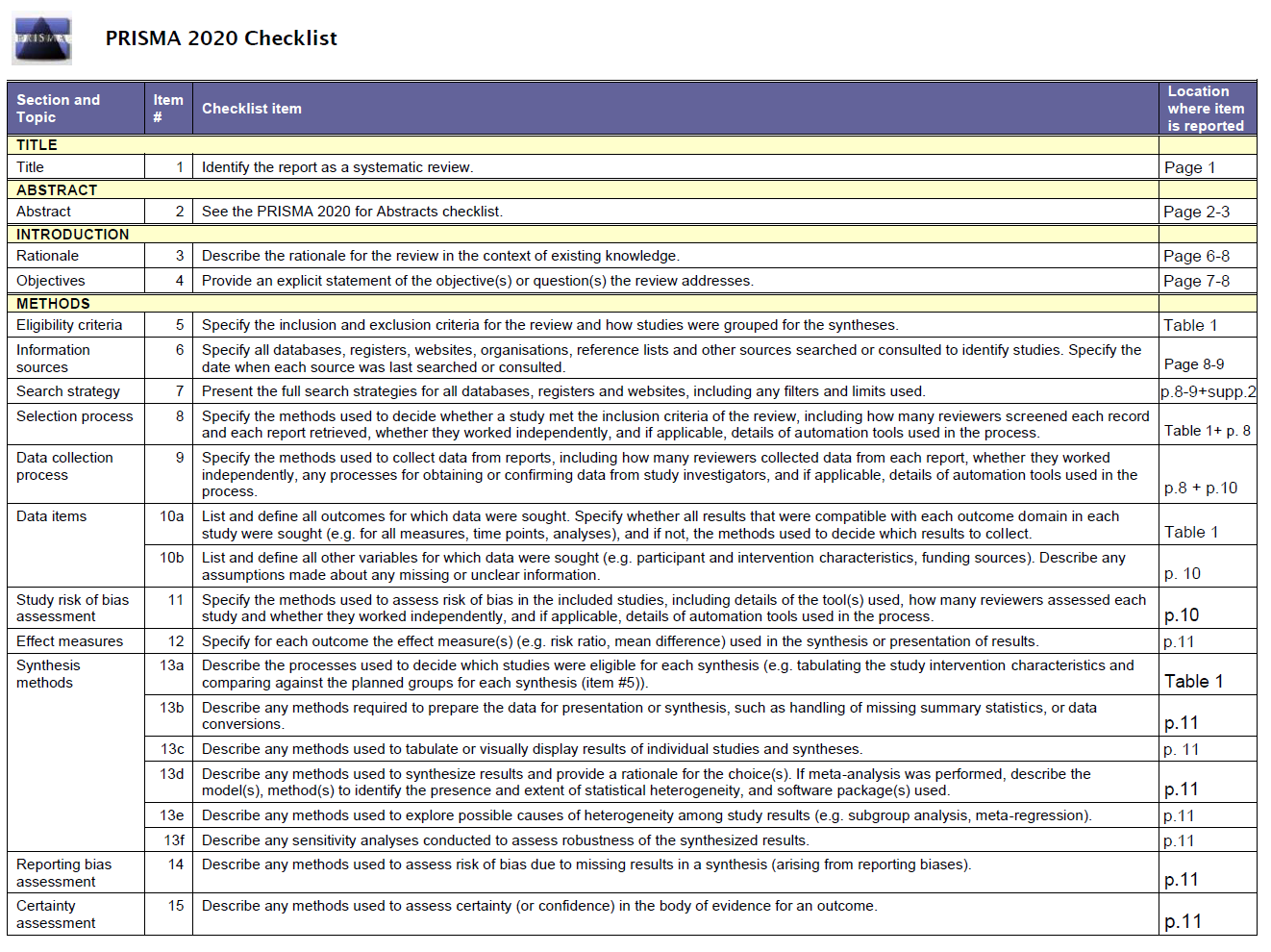

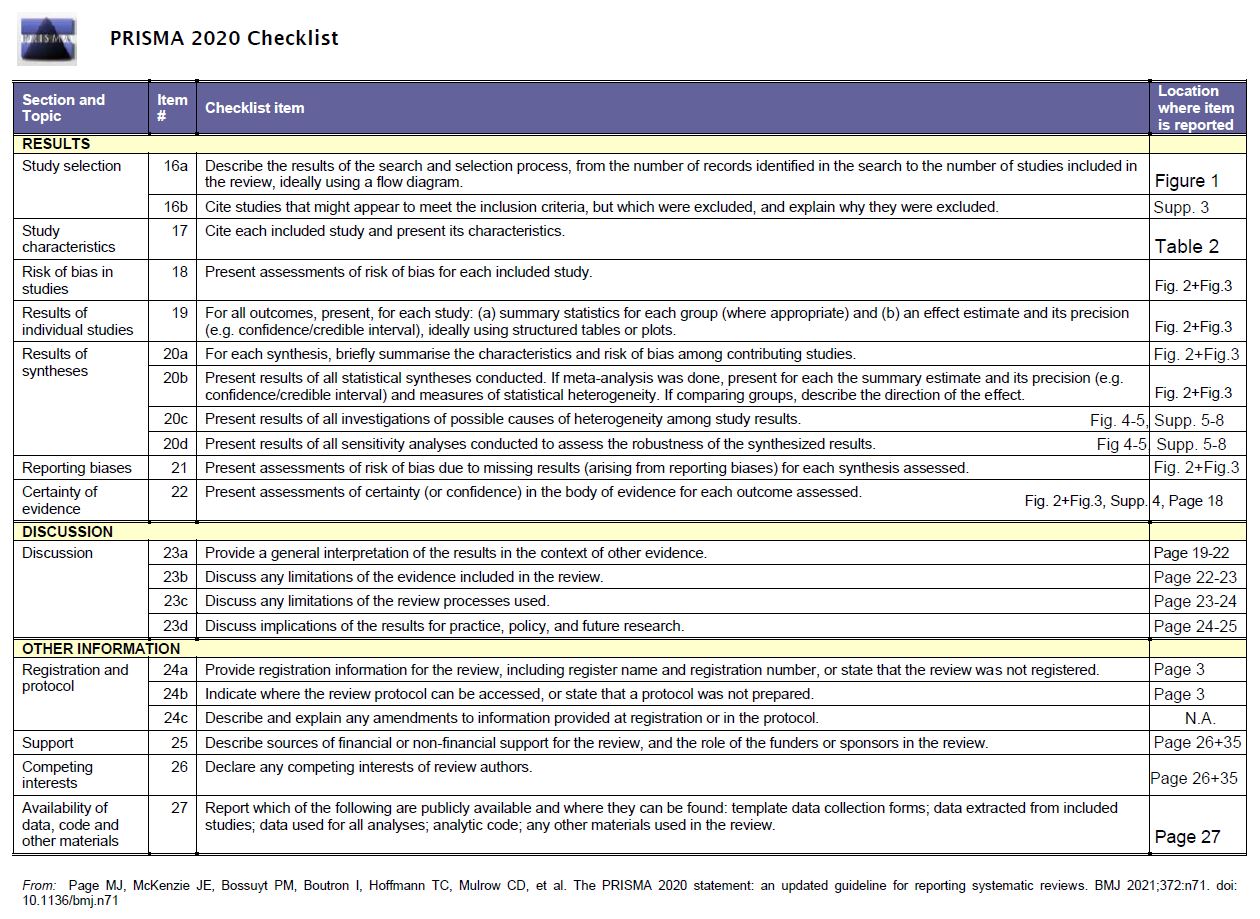


**Supplementary file S2**

**Databases, search strategy and number of articles retrieved from the database searches**

| **Database** | **Search key** | **Date** | **Hits** |
| --- | --- | --- | --- |
| PubMed | (#1 OR #3) AND (#2 OR #4)  #1 = "chronic pain"[MeSH Terms] OR "musculoskeletal pain"[MeSH Terms]   #2 = “placebos”[MeSH Terms]  #3= "long lasting pain"[Title/Abstract] OR "intermittent pain"[Title/Abstract] OR "long term pain"[Title/Abstract] OR "persistent pain"[Title/Abstract] OR "intractable pain"[Title/Abstract] OR "nociceptive pain"[Title/Abstract] OR "generalized pain"[Title/Abstract] OR "widespread pain"[Title/Abstract] OR "fibromyalgia"[Title/Abstract] OR "myofascial pain syndrome"[Title/Abstract] OR "myalgia"[Title/Abstract] OR "idiopathic pain"[Title/Abstract] OR "diffuse pain"[Title/Abstract] OR “aspecific pain”[ Title/Abstract] OR "non specific pain"[Title/Abstract] OR "nonspecific pain"[Title/Abstract] OR "musculoskeletal pain syndrome"[Title/Abstract] OR "chronic pain syndrome"[Title/Abstract] OR "somatoform pain"[Title/Abstract] OR "non cancer pain"[Title/Abstract] OR "non malignant pain"[Title/Abstract] OR "benign pain"[Title/Abstract] OR "back pain"[Title/Abstract] OR "low back pain"[Title/Abstract] OR "neck pain"[Title/Abstract] OR "shoulder pain"[Title/Abstract]  #4 = "open label placebo*"[Title/Abstract] OR "open placebo*"[Title/Abstract] OR "placebo response"[Title/Abstract] OR "ethical use of placebo*"[Title/Abstract] OR "no deceptive placebo*"[Title/Abstract] OR "non decept"[Title/Abstract] OR "nonconceal"[Title/Abstract] OR "non conceal"[Title/Abstract] OR "nonblind"[Title/Abstract] OR "non blind"[Title/Abstract] OR "without deception"[Title/Abstract] OR "without conceal"[Title/Abstract] OR "without blind"[Title/Abstract] OR "sugar pill*"[Title/Abstract] OR "placebo creme*"[Title/Abstract] OR "placebo pill*"[Title/Abstract] | 25/09/2023 ------------- 28/08/2024 | 392 --------- 2 |
| Web of Science | (#1) AND (#2) #1 TS=("chronic pain" OR "musculoskeletal pain" OR "long lasting pain" OR "intermittent pain" OR "long term pain" OR "persistent pain" OR "intractable pain" OR "nociceptive pain" OR "generalized pain" OR "widespread pain" OR "fibromyalgia" OR "myofascial pain syndrome" OR "myalgia" OR "idiopathic pain" OR "diffuse pain" OR “aspecific pain” OR "non specific pain" OR "nonspecific pain" OR "musculoskeletal pain syndrome" OR "chronic pain syndrome" OR "somatoform pain" OR "non cancer pain" OR "non malignant pain" OR "benign pain" OR "back pain" OR "low back pain" OR "neck pain" OR "shoulder pain")  #2 TS=("open label placebo*" OR "open placebo*" OR "placebo response" OR "ethical use of placebo*" OR "no deceptive placebo*" OR "non decept" OR "nonconceal" OR "non conceal" OR "nonblind" OR "non blind" OR "without deception" OR "without conceal" OR "without blind" OR "sugar pill*" OR "placebo creme*" OR "placebo pill*") | 25/09/2023 ------------- 28/08/2024 | 204 --------- 9 |
| PsycINFO | (#1 OR #2) AND (#3 OR #4) #1 TI "chronic pain" OR "musculoskeletal pain" OR "long lasting pain" OR "intermittent pain" OR "long term pain" OR "persistent pain" OR "intractable pain" OR "nociceptive pain" OR "generalized pain" OR "widespread pain" OR "fibromyalgia" OR "myofascial pain syndrome" OR "myalgia" OR "idiopathic pain" OR "diffuse pain" OR “aspecific pain” OR "non specific pain" OR "nonspecific pain" OR "musculoskeletal pain syndrome" OR "chronic pain syndrome" OR "somatoform pain" OR "non cancer pain" OR "non malignant pain" OR "benign pain" OR "back pain" OR "low back pain" OR "neck pain" OR "shoulder pain" #2 AB "chronic pain" OR "musculoskeletal pain" OR "long lasting pain" OR "intermittent pain" OR "long term pain" OR "persistent pain" OR "intractable pain" OR "nociceptive pain" OR "generalized pain" OR "widespread pain" OR "fibromyalgia" OR "myofascial pain syndrome" OR "myalgia" OR "idiopathic pain" OR "diffuse pain" OR “aspecific pain” OR "non specific pain" OR "nonspecific pain" OR "musculoskeletal pain syndrome" OR "chronic pain syndrome" OR "somatoform pain" OR "non cancer pain" OR "non malignant pain" OR "benign pain" OR "back pain" OR "low back pain" OR "neck pain" OR "shoulder pain"  #3 TI “placebo*” OR "open label placebo*" OR "open placebo*" OR "placebo response" OR "placebo effect*" OR "ethical use of placebo*" OR "no deceptive placebo*" OR "non decept" OR "nonconceal" OR "non conceal" OR "nonblind" OR "non blind" OR "without deception" OR "without conceal" OR "without blind" OR "sugar pill*" OR "placebo creme*" OR "placebo pill*"  #4 AB “placebo*” OR "open label placebo*" OR "open placebo*" OR "placebo response" OR "placebo effect*" OR "ethical use of placebo*" OR "no deceptive placebo*" OR "non decept" OR "nonconceal" OR "non conceal" OR "nonblind" OR "non blind" OR "without deception" OR "without conceal" OR "without blind" OR "sugar pill*" OR "placebo creme*" OR "placebo pill*" | 25/09/2023 ------------- 28/08/2024 | 960 --------- 11 |
| EMBASE | (#1) AND (#2) #1 TS= "chronic pain" OR "musculoskeletal pain" OR "long lasting pain" OR "intermittent pain" OR "long term pain" OR "persistent pain" OR "intractable pain" OR "nociceptive pain" OR "generalized pain" OR "widespread pain" OR "fibromyalgia" OR "myofascial pain syndrome" OR "myalgia" OR "idiopathic pain" OR "diffuse pain" OR “aspecific pain” OR "non specific pain" OR "nonspecific pain" OR "musculoskeletal pain syndrome" OR "chronic pain syndrome" OR "somatoform pain" OR "non cancer pain" OR "non malignant pain" OR "benign pain" OR "back pain" OR "low back pain" OR "neck pain" OR "shoulder pain"  #2 TS= "open label placebo*" OR "open placebo*" OR "placebo response" OR "ethical use of placebo*" OR "no deceptive placebo*" OR "non decept" OR "nonconceal" OR "non conceal" OR "nonblind" OR "non blind" OR "without deception" OR "without conceal" OR "without blind" OR "sugar pill*" OR "placebo creme*" OR "placebo pill*" | 25/09/2023 ------------- 28/08/2024 | 176 --------- 21 |

**Supplementary file S3**


**Excluded Studies and Reasons for Exclusion**

| **Ref.** | **First Author, Year** | **Journal** | **Reasons(s) for exclusion** |
| --- | --- | --- | --- |
| ^1^ | Carvalho, 2021 | *PAIN* | Wrong study design |
| ^2^ | De Vries, 2016 | *Scandinavian Journal of Medicine & Science in Sports* | Wrong intervention |
| ^3^ | Emadi Andani, 2024 | *British Journal of Psychology* | Wrong population |
| ^4^ | Friehs, 2024 | *European Journal of Pain* | Wrong intervention |
| ^5^ | Grotle, 2017 | *Journal of Physiotherapy* | Wrong study design |
| ^6^ | Henriksen, 2023 | *Osteoarthritis and Cartilage* | Wrong study design |
| ^7^ | Klinger, 2017 | *PAIN* | Wrong intervention |
| ^8^ | Leichtfried, 2014 | *Pain Medicine* | Wrong intervention |
| ^9^ | Petersen, 2014 | *PAIN* | Wrong population |

**Intervention (n = 4)
Study design (n = 3)
Population (n = 2)**


References

1. Carvalho C, Pais M, Cunha L, Rebouta P, Kaptchuk TJ, Kirsch I. Open-label placebo for chronic low back pain: a 5-year follow-up. *Pain*. 2021;162(5):1521-1527. doi:10.1097/j.pain.0000000000002162

2. De Vries A, Zwerver J, Diercks R, et al. Effect of patellar strap and sports tape on pain in patellar tendinopathy: A randomized controlled trial. *Scand J Med Sci Sports*. 2016;26(10):1217-1224. doi:10.1111/sms.12556

3. Emadi Andani M, Barbiani D, Bonetto M, Menegaldo R, Villa‐Sánchez B, Fiorio M. Preserving the placebo effect after disclosure: A new perspective on non‐deceptive placebos. *Br J Psychol*. 2024;115(3):437-453. doi:10.1111/bjop.12696

4. Friehs T, Milde C, Glombiewski JA, Kube T. Change in pain expectations but no open‐label placebo analgesia: An experimental study using the heat pain paradigm. *Eur J Pain*. 2024;28(5):769-785. doi:10.1002/ejp.2216

5. Grotle M, Hagen KB. Placebo pills provided without deception may help to reduce pain and disability in people with chronic low back pain [synopsis]. *J Physiother*. 2017;63(3):183. doi:10.1016/j.jphys.2017.05.002

6. Henriksen M, Nielsen SM, Christensen R, et al. Who are likely to benefit from the Good Life with osteoArthritis in Denmark (GLAD) exercise and education program? An effect modifier analysis of a randomised controlled trial. *Osteoarthritis Cartilage*. 2023;31(1):106-114. doi:10.1016/j.joca.2022.09.001

7. Klinger R, Kothe R, Schmitz J, Kamping S, Flor H. Placebo effects of a sham opioid solution: a randomized controlled study in patients with chronic low back pain. *Pain*. 2017;158(10):1893-1902. doi:10.1097/j.pain.0000000000000977

8. Leichtfried V, Matteucci Gothe R, Kantner-Rumplmair W, et al. Short-Term Effects of Bright Light Therapy in Adults with Chronic Nonspecific Back Pain: A Randomized Controlled Trial. *Pain Med*. 2014;15(12):2003-2012. doi:10.1111/pme.12503

9. Petersen GL, Finnerup NB, Grosen K, et al. Expectations and positive emotional feelings accompany reductions in ongoing and evoked neuropathic pain following placebo interventions. *Pain*. 2014;155(12):2687-2698. doi:10.1016/j.pain.2014.09.036

**Supplementary file S4**
**GRADE assessments**

|  | Domains | Criteria | Outcome Tests of Physical Function | Outcome Physical functioning | Outcome Pain |
| --- | --- | --- | --- | --- | --- |
| Downgrading | Limitations in study design | The level of evidence is downgraded when at least one study scores a “High RoB” (red) on at least one domain, or if a study has the majority of the domains is classified as “Moderate RoB” (Yellow). | NOT DOWNGRADED  No “red” domains. | DOWNGRADED  Several studies had one or more “red” domains. | DOWNGRADED  Several studies had one or more “red” domains. |
|  | Inconsistency in results | The level of evidence is downgraded if “substantial” or “considerable” heterogeneity (*I*^2^ ≥75%) is present. Moreover, the level of evidence is downgraded if the direction of the estimates is inconsistent (positive, null, or negative results), or when there is little overlap of the 95% confidence intervals of the included studies. |  | NOT DOWNGRADED  The estimates largely overlapped each other and showed similar point estimates. Moreover, *I*^2^ = 0% | NOT DOWNGRADED  The estimates largely overlapped each other and showed similar point estimates. Moreover, *I*^2^ = 0% |
|  | Indirectness of evidence | The level of the evidence is downgraded if one or more parts of the PICO do not correspond to the research question or clinical setting. | NOT DOWNGRADED  The studies were similar in population, intervention, control groups, and outcomes. | NOT DOWNGRADED  The studies were similar in population, intervention, control groups, and outcomes. | NOT DOWNGRADED  The studies were similar in population, intervention, control groups, and outcomes. |
|  | Imprecision | The level of the evidence is downgraded when the 95% confidence intervals around the effect estimate are wide and there is no clear estimate of the effect. | DOWNGRADED  The 95% confidence intervals of the estimates in the studies were wide. | DOWNGRADED  The 95% confidence intervals of the estimates in the studies included and the overall point estimate were wide. | DOWNGRADED  The 95% confidence intervals of the estimates in the studies included and the overall point estimate were wide. |
|  | Publication bias | The level of the evidence is downgraded if searches in trial registries or grey literature detect studies that are not available in the databases. Moreover, if more than 10 studies are available, the level of the evidence is also downgraded if visual analyses of the funnel plots show skewed distributions and Egger’s tests show significant results (p<0.05). | NOT DOWNGRADED  No additional studies were found in trial registries or grey literature. | NOT DOWNGRADED  No additional studies were found in trial registries or grey literature. | NOT DOWNGRADED  No additional studies were found in trial registries or grey literature. |
| Upgrading | Magnitudes of effects | The level of the evidence is upgraded when the effects are large; -0.8 < SMD > 0.8 with small 95%Cis. | NOT UPGRADED  No effects were found. | NOT UPGRADED  Small effects were found. | NOT UPGRADED  Small effects were found. |
|  | Dose-response gradient | The level of the evidence is upgraded when there is a clear dose-response relationship. | N/A | NOT UPGRADED  No clear dose-response relationship. | NOT UPGRADED  No clear dose-response relationship. |
|  | Plausible confounding | N/A | N/A | N/A | N/A |

**Supplementary file S5**

**Correlation between publication year and effect size for PROMs of physical function**


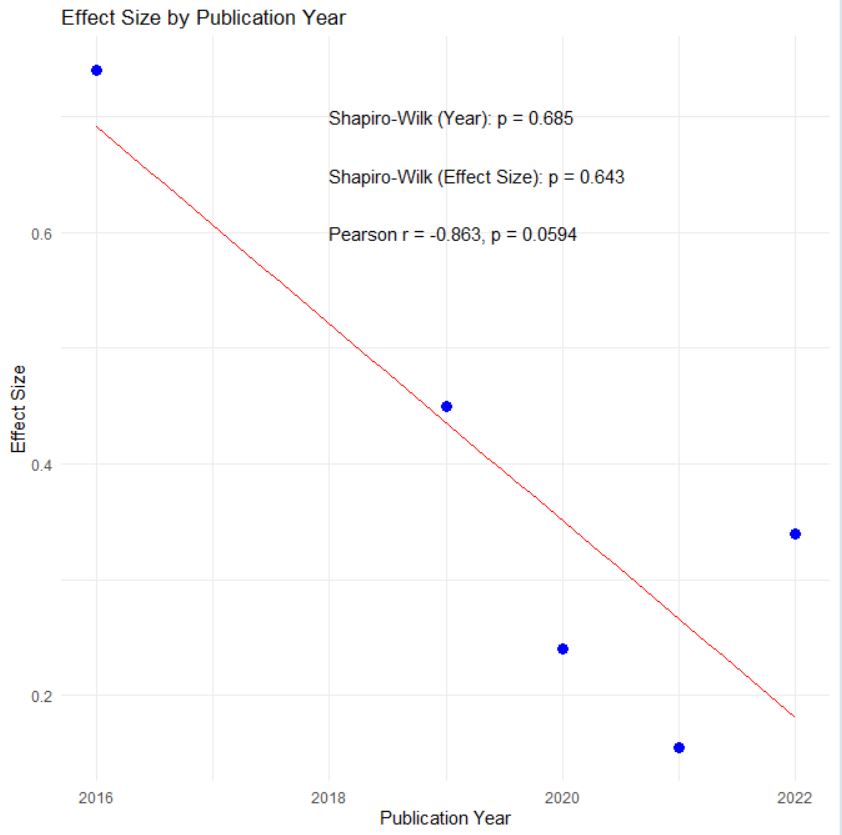


**Supplementary file S6**

**Correlation between publication year and effect size for PROMs of pain intensity**


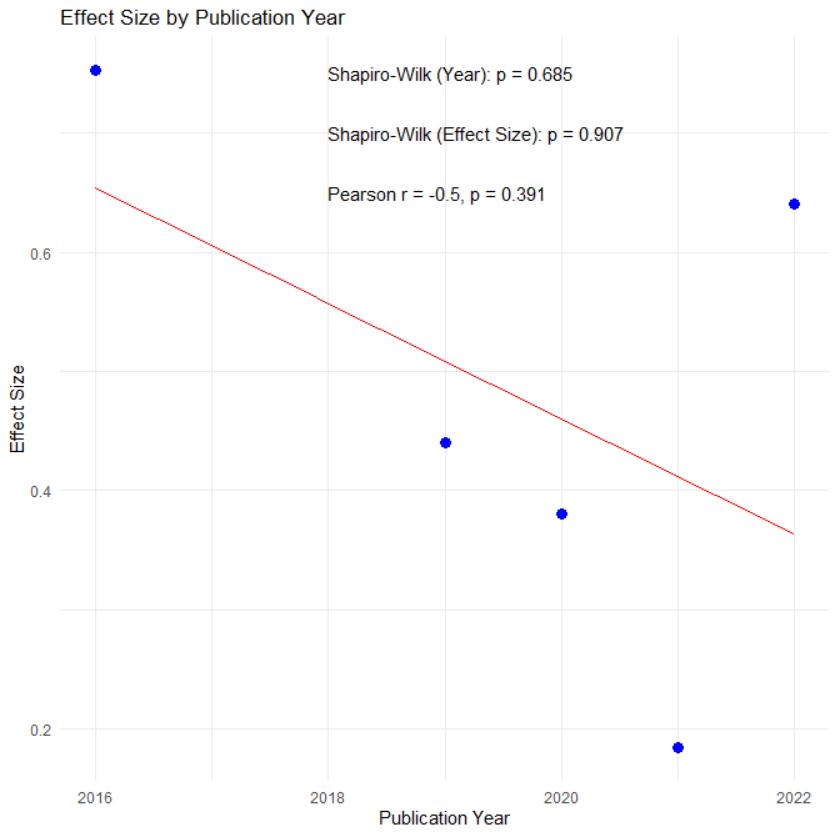


**Supplementary file S7**

**Correlation between sample size and effect size for PROMS of physical function**


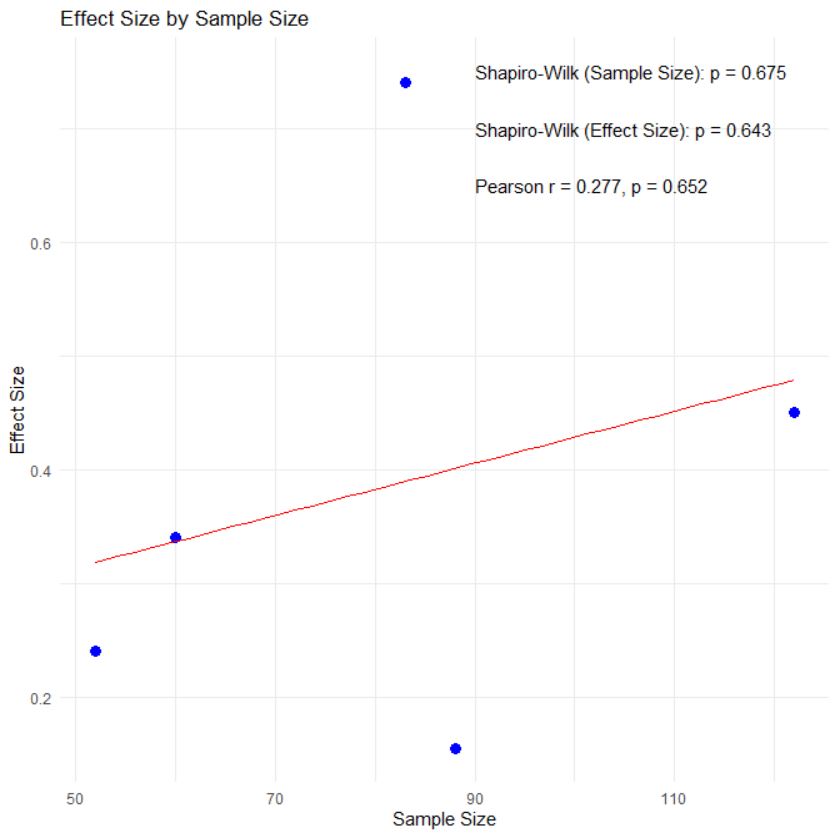


**Supplementary file S8**

**Correlation between sample size and effect size for PROMS of pain intensity**


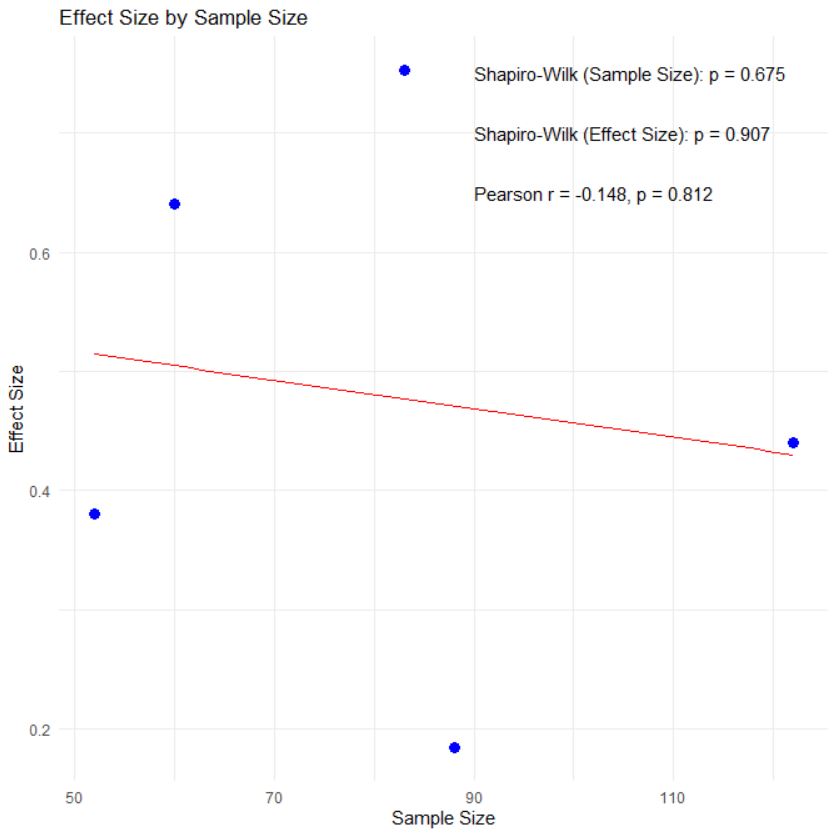

Supplement: Supplementary file 1 — Supplementary Material 1 [file 41598_2025_9415_MOESM1_ESM.docx]
