# Supplementary figures and images for: A systematic review and meta analysis of open label placebo effects in chronic musculoskeletal pain
Source: Sci Rep. 2025 Jul 5;15:24007. doi: 10.1038/s41598-025-09415-y (PMC12228692; doi:10.1038/s41598-025-09415-y)

**Supplement S7** – **Correlation between sample size and effect size for PROMs of physical function**
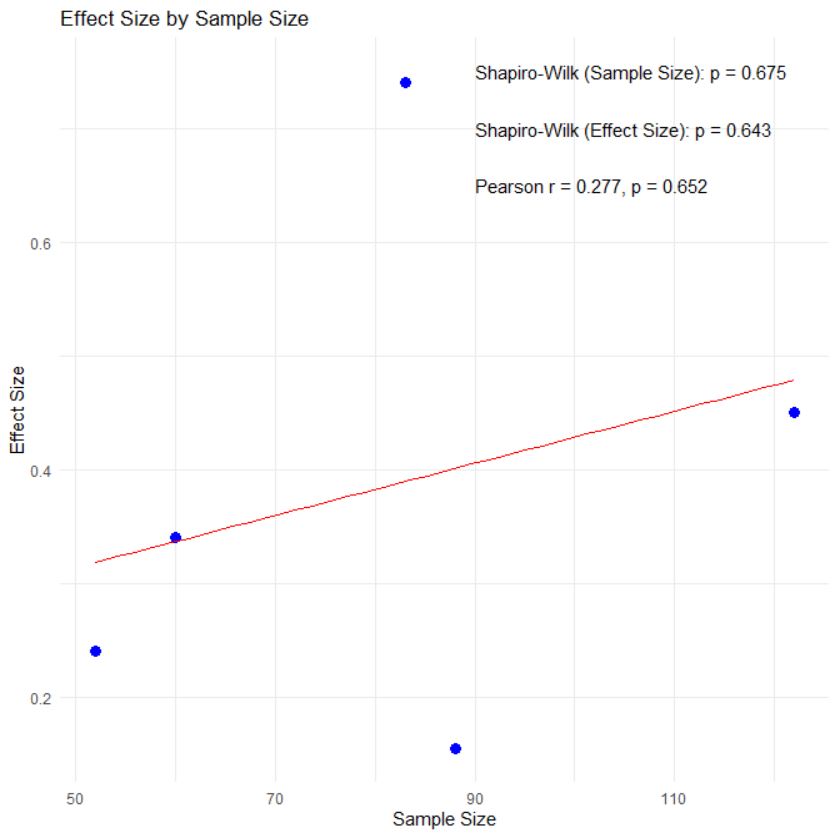

Supplement: Supplementary file 2 — Supplementary Material 2 [file 41598_2025_9415_MOESM2_ESM.docx]

**Supplement S8** – **Correlation between sample size and effect size for PROMs of pain intensity**
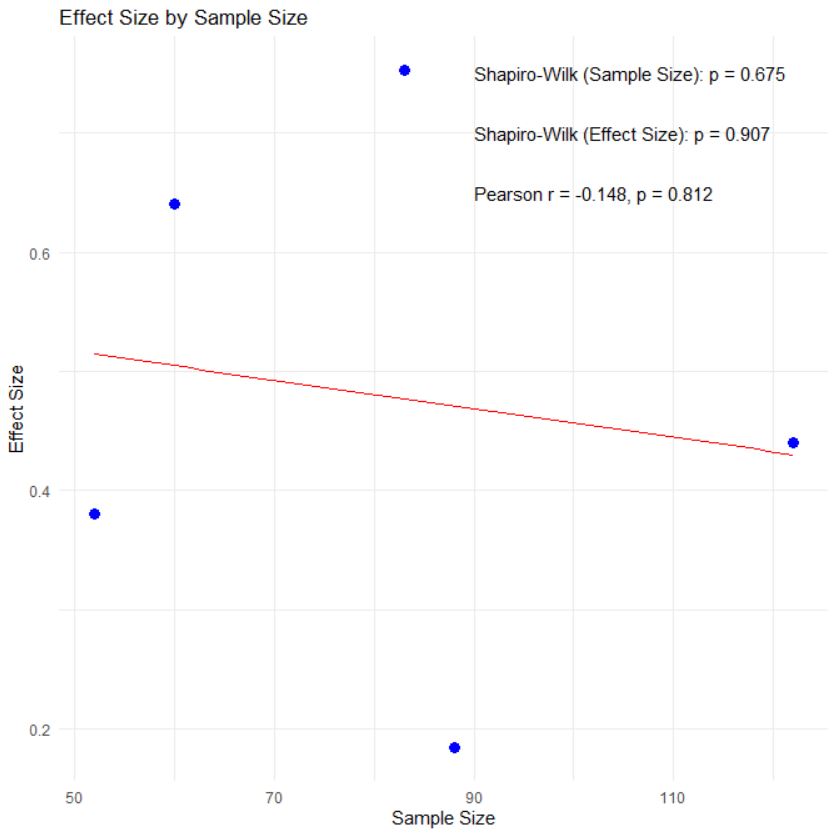

Supplement: Supplementary file 6 — Supplementary Material 6 [file 41598_2025_9415_MOESM6_ESM.docx]
