# Supplementary material for: A systematic review and meta analysis of open label placebo effects in chronic musculoskeletal pain
Source: Sci Rep. 2025 Jul 5;15:24007. doi: 10.1038/s41598-025-09415-y (PMC12228692; doi:10.1038/s41598-025-09415-y)

**Supplement S5** – **Correlation between publication year and effect size for PROMs of physical function**


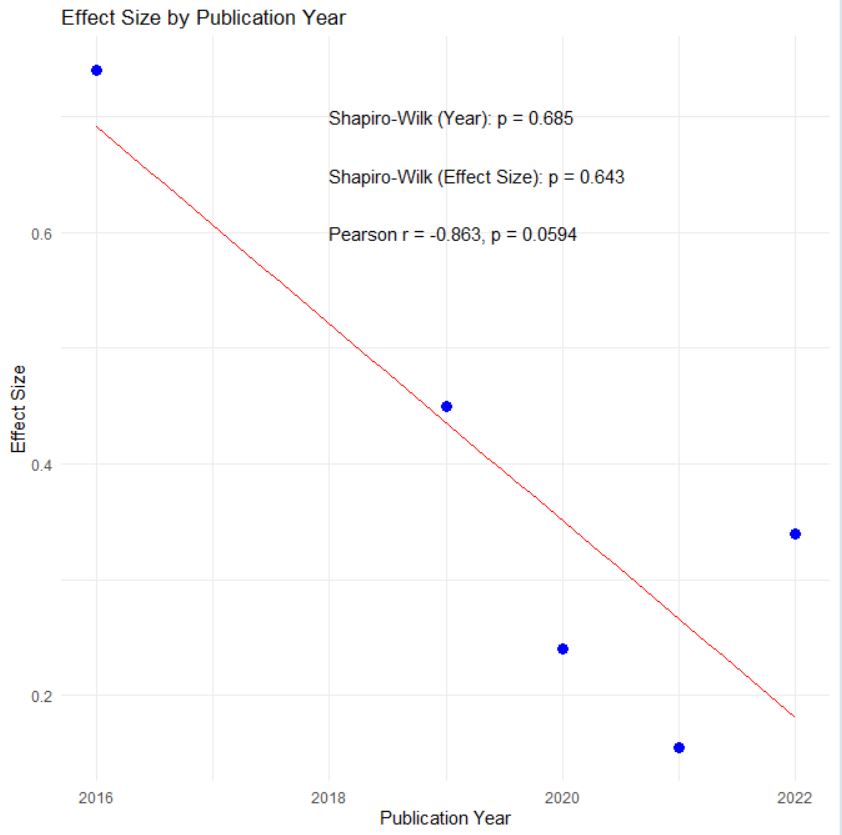

Supplement: Supplementary file 3 — Supplementary Material 3 [file 41598_2025_9415_MOESM3_ESM.docx]
