# Supplementary material for: A systematic review and meta analysis of open label placebo effects in chronic musculoskeletal pain
Source: Sci Rep. 2025 Jul 5;15:24007. doi: 10.1038/s41598-025-09415-y (PMC12228692; doi:10.1038/s41598-025-09415-y)
Supplement: Supplementary file 4 — Supplementary Material 4 [file 41598_2025_9415_MOESM4_ESM.docx]

**Supplement S4** – **GRADE assessments**

|  | Domains | Criteria | Outcome Tests of Physical Function | Outcome Physical functioning | Outcome Pain |
| --- | --- | --- | --- | --- | --- |
| Downgrading | Limitations in study design | The level of evidence is downgraded when at least one study scores a “High RoB” (red) on at least one domain, or if a study has the majority of the domains is classified as “Moderate RoB” (Yellow). | NOT DOWNGRADED  No “red” domains. | DOWNGRADED  Several studies had one or more “red” domains. | DOWNGRADED  Several studies had one or more “red” domains. |
|  | Inconsistency in results | The level of evidence is downgraded if “substantial” or “considerable” heterogeneity (*I*^2^ ≥75%) is present. Moreover, the level of evidence is downgraded if the direction of the estimates is inconsistent (positive, null, or negative results), or when there is little overlap of the 95% confidence intervals of the included studies. |  | NOT DOWNGRADED  The estimates largely overlapped each other and showed similar point estimates. Moreover, *I*^2^ = 0% | NOT DOWNGRADED  The estimates largely overlapped each other and showed similar point estimates. Moreover, *I*^2^ = 0% |
|  | Indirectness of evidence | The level of the evidence is downgraded if one or more parts of the PICO do not correspond to the research question or clinical setting. | NOT DOWNGRADED  The studies were similar in population, intervention, control groups, and outcomes. | NOT DOWNGRADED  The studies were similar in population, intervention, control groups, and outcomes. | NOT DOWNGRADED  The studies were similar in population, intervention, control groups, and outcomes. |
|  | Imprecision | The level of the evidence is downgraded when the 95% confidence intervals around the effect estimate are wide and there is no clear estimate of the effect. | DOWNGRADED  The 95% confidence intervals of the estimates in the studies were wide. | DOWNGRADED  The 95% confidence intervals of the estimates in the studies included and the overall point estimate were wide. | DOWNGRADED  The 95% confidence intervals of the estimates in the studies included and the overall point estimate were wide. |
|  | Publication bias | The level of the evidence is downgraded if searches in trial registries or grey literature detect studies that are not available in the databases. Moreover, if more than 10 studies are available, the level of the evidence is also downgraded if visual analyses of the funnel plots show skewed distributions and Egger’s tests show significant results (p<0.05). | NOT DOWNGRADED  No additional studies were found in trial registries or grey literature. | NOT DOWNGRADED  No additional studies were found in trial registries or grey literature. | NOT DOWNGRADED  No additional studies were found in trial registries or grey literature. |
| Upgrading | Magnitudes of effects | The level of the evidence is upgraded when the effects are large; -0.8 < SMD > 0.8 with small 95%Cis. | NOT UPGRADED  No effects were found. | NOT UPGRADED  Small effects were found. | NOT UPGRADED  Small effects were found. |
|  | Dose-response gradient | The level of the evidence is upgraded when there is a clear dose-response relationship. | N/A | NOT UPGRADED  No clear dose-response relationship. | NOT UPGRADED  No clear dose-response relationship. |
|  | Plausible confounding | N/A | N/A | N/A | N/A |
