# Supplementary material for: A systematic review and meta analysis of open label placebo effects in chronic musculoskeletal pain
Source: Sci Rep. 2025 Jul 5;15:24007. doi: 10.1038/s41598-025-09415-y (PMC12228692; doi:10.1038/s41598-025-09415-y)

**Supplement S6** – **Correlation between publication year and effect size for PROMs of pain intensity**
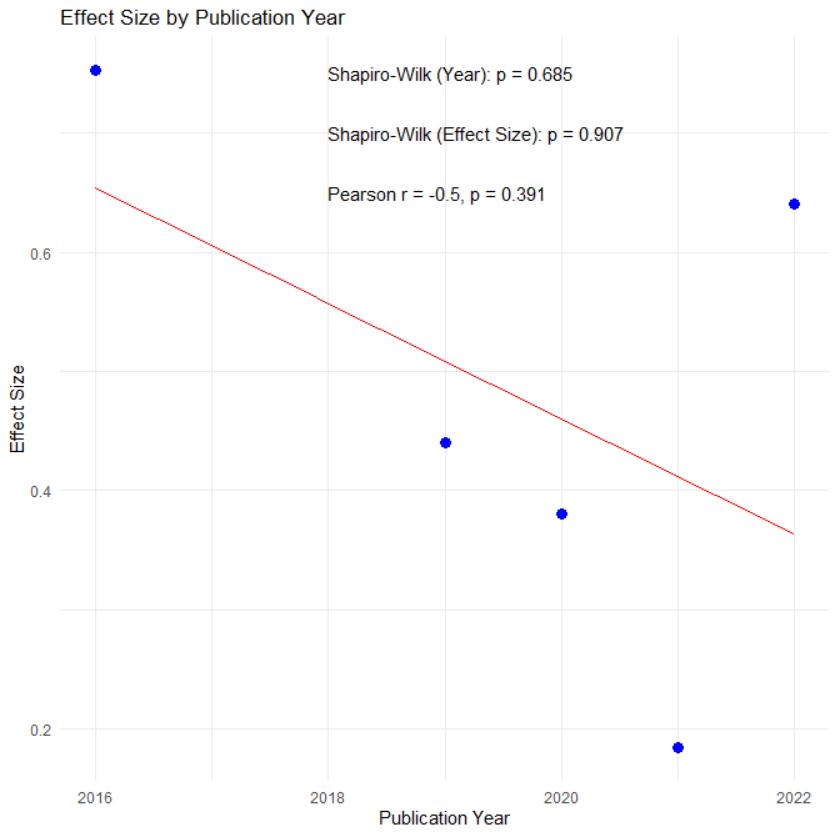

Supplement: Supplementary file 5 — Supplementary Material 5 [file 41598_2025_9415_MOESM5_ESM.docx]
