# Supplementary material for: A systematic review and meta analysis of open label placebo effects in chronic musculoskeletal pain
Source: Sci Rep. 2025 Jul 5;15:24007. doi: 10.1038/s41598-025-09415-y (PMC12228692; doi:10.1038/s41598-025-09415-y)
Supplement: Supplementary file 7 — Supplementary Material 7 [file 41598_2025_9415_MOESM7_ESM.docx]

**Supplement S3** – **Excluded Studies and Reasons for Exclusion**

| **Ref.** | **First Author, Year** | **Journal** | **Reasons(s) for exclusion** |
| --- | --- | --- | --- |
| ^1^ | Carvalho, 2021 | *PAIN* | Wrong study design |
| ^2^ | De Vries, 2016 | *Scandinavian Journal of Medicine & Science in Sports* | Wrong intervention |
| ^3^ | Emadi Andani, 2024 | *British Journal of Psychology* | Wrong population |
| ^4^ | Friehs, 2024 | *European Journal of Pain* | Wrong intervention |
| ^5^ | Grotle, 2017 | *Journal of Physiotherapy* | Wrong study design |
| ^6^ | Henriksen, 2023 | *Osteoarthritis and Cartilage* | Wrong study design |
| ^7^ | Klinger, 2017 | *PAIN* | Wrong intervention |
| ^8^ | Leichtfried, 2014 | *Pain Medicine* | Wrong intervention |
| ^9^ | Petersen, 2014 | *PAIN* | Wrong population |

**Intervention (n = 4)
Study design (n = 3)
Population (n = 2)**


References

1. Carvalho C, Pais M, Cunha L, Rebouta P, Kaptchuk TJ, Kirsch I. Open-label placebo for chronic low back pain: a 5-year follow-up. *Pain*. 2021;162(5):1521-1527. doi:10.1097/j.pain.0000000000002162

2. De Vries A, Zwerver J, Diercks R, et al. Effect of patellar strap and sports tape on pain in patellar tendinopathy: A randomized controlled trial. *Scand J Med Sci Sports*. 2016;26(10):1217-1224. doi:10.1111/sms.12556

3. Emadi Andani M, Barbiani D, Bonetto M, Menegaldo R, Villa‐Sánchez B, Fiorio M. Preserving the placebo effect after disclosure: A new perspective on non‐deceptive placebos. *Br J Psychol*. 2024;115(3):437-453. doi:10.1111/bjop.12696

4. Friehs T, Milde C, Glombiewski JA, Kube T. Change in pain expectations but no open‐label placebo analgesia: An experimental study using the heat pain paradigm. *Eur J Pain*. 2024;28(5):769-785. doi:10.1002/ejp.2216

5. Grotle M, Hagen KB. Placebo pills provided without deception may help to reduce pain and disability in people with chronic low back pain [synopsis]. *J Physiother*. 2017;63(3):183. doi:10.1016/j.jphys.2017.05.002

6. Henriksen M, Nielsen SM, Christensen R, et al. Who are likely to benefit from the Good Life with osteoArthritis in Denmark (GLAD) exercise and education program? An effect modifier analysis of a randomised controlled trial. *Osteoarthritis Cartilage*. 2023;31(1):106-114. doi:10.1016/j.joca.2022.09.001

7. Klinger R, Kothe R, Schmitz J, Kamping S, Flor H. Placebo effects of a sham opioid solution: a randomized controlled study in patients with chronic low back pain. *Pain*. 2017;158(10):1893-1902. doi:10.1097/j.pain.0000000000000977

8. Leichtfried V, Matteucci Gothe R, Kantner-Rumplmair W, et al. Short-Term Effects of Bright Light Therapy in Adults with Chronic Nonspecific Back Pain: A Randomized Controlled Trial. *Pain Med*. 2014;15(12):2003-2012. doi:10.1111/pme.12503

9. Petersen GL, Finnerup NB, Grosen K, et al. Expectations and positive emotional feelings accompany reductions in ongoing and evoked neuropathic pain following placebo interventions. *Pain*. 2014;155(12):2687-2698. doi:10.1016/j.pain.2014.09.036
