# Supplementary material for: A systematic review and meta analysis of open label placebo effects in chronic musculoskeletal pain
Source: Sci Rep. 2025 Jul 5;15:24007. doi: 10.1038/s41598-025-09415-y (PMC12228692; doi:10.1038/s41598-025-09415-y)
Supplement: Supplementary file 8 — Supplementary Material 8 [file 41598_2025_9415_MOESM8_ESM.docx]

**Supplement S2** – **Databases, search strategy and number of articles retrieved from the database searches**

| **Database** | **Search key** | **Date** | **Hits** |
| --- | --- | --- | --- |
| PubMed | (#1 OR #3) AND (#2 OR #4)  #1 = "chronic pain"[MeSH Terms] OR "musculoskeletal pain"[MeSH Terms]   #2 = “placebos”[MeSH Terms]  #3= "long lasting pain"[Title/Abstract] OR "intermittent pain"[Title/Abstract] OR "long term pain"[Title/Abstract] OR "persistent pain"[Title/Abstract] OR "intractable pain"[Title/Abstract] OR "nociceptive pain"[Title/Abstract] OR "generalized pain"[Title/Abstract] OR "widespread pain"[Title/Abstract] OR "fibromyalgia"[Title/Abstract] OR "myofascial pain syndrome"[Title/Abstract] OR "myalgia"[Title/Abstract] OR "idiopathic pain"[Title/Abstract] OR "diffuse pain"[Title/Abstract] OR “aspecific pain”[ Title/Abstract] OR "non specific pain"[Title/Abstract] OR "nonspecific pain"[Title/Abstract] OR "musculoskeletal pain syndrome"[Title/Abstract] OR "chronic pain syndrome"[Title/Abstract] OR "somatoform pain"[Title/Abstract] OR "non cancer pain"[Title/Abstract] OR "non malignant pain"[Title/Abstract] OR "benign pain"[Title/Abstract] OR "back pain"[Title/Abstract] OR "low back pain"[Title/Abstract] OR "neck pain"[Title/Abstract] OR "shoulder pain"[Title/Abstract]  #4 = "open label placebo*"[Title/Abstract] OR "open placebo*"[Title/Abstract] OR "placebo response"[Title/Abstract] OR "ethical use of placebo*"[Title/Abstract] OR "no deceptive placebo*"[Title/Abstract] OR "non decept"[Title/Abstract] OR "nonconceal"[Title/Abstract] OR "non conceal"[Title/Abstract] OR "nonblind"[Title/Abstract] OR "non blind"[Title/Abstract] OR "without deception"[Title/Abstract] OR "without conceal"[Title/Abstract] OR "without blind"[Title/Abstract] OR "sugar pill*"[Title/Abstract] OR "placebo creme*"[Title/Abstract] OR "placebo pill*"[Title/Abstract] | 25/09/2023 ------------- 28/08/2024 | 392 --------- 2 |
| Web of Science | (#1) AND (#2) #1 TS=("chronic pain" OR "musculoskeletal pain" OR "long lasting pain" OR "intermittent pain" OR "long term pain" OR "persistent pain" OR "intractable pain" OR "nociceptive pain" OR "generalized pain" OR "widespread pain" OR "fibromyalgia" OR "myofascial pain syndrome" OR "myalgia" OR "idiopathic pain" OR "diffuse pain" OR “aspecific pain” OR "non specific pain" OR "nonspecific pain" OR "musculoskeletal pain syndrome" OR "chronic pain syndrome" OR "somatoform pain" OR "non cancer pain" OR "non malignant pain" OR "benign pain" OR "back pain" OR "low back pain" OR "neck pain" OR "shoulder pain")  #2 TS=("open label placebo*" OR "open placebo*" OR "placebo response" OR "ethical use of placebo*" OR "no deceptive placebo*" OR "non decept" OR "nonconceal" OR "non conceal" OR "nonblind" OR "non blind" OR "without deception" OR "without conceal" OR "without blind" OR "sugar pill*" OR "placebo creme*" OR "placebo pill*") | 25/09/2023 ------------- 28/08/2024 | 204 --------- 9 |
| PsycINFO | (#1 OR #2) AND (#3 OR #4) #1 TI "chronic pain" OR "musculoskeletal pain" OR "long lasting pain" OR "intermittent pain" OR "long term pain" OR "persistent pain" OR "intractable pain" OR "nociceptive pain" OR "generalized pain" OR "widespread pain" OR "fibromyalgia" OR "myofascial pain syndrome" OR "myalgia" OR "idiopathic pain" OR "diffuse pain" OR “aspecific pain” OR "non specific pain" OR "nonspecific pain" OR "musculoskeletal pain syndrome" OR "chronic pain syndrome" OR "somatoform pain" OR "non cancer pain" OR "non malignant pain" OR "benign pain" OR "back pain" OR "low back pain" OR "neck pain" OR "shoulder pain" #2 AB "chronic pain" OR "musculoskeletal pain" OR "long lasting pain" OR "intermittent pain" OR "long term pain" OR "persistent pain" OR "intractable pain" OR "nociceptive pain" OR "generalized pain" OR "widespread pain" OR "fibromyalgia" OR "myofascial pain syndrome" OR "myalgia" OR "idiopathic pain" OR "diffuse pain" OR “aspecific pain” OR "non specific pain" OR "nonspecific pain" OR "musculoskeletal pain syndrome" OR "chronic pain syndrome" OR "somatoform pain" OR "non cancer pain" OR "non malignant pain" OR "benign pain" OR "back pain" OR "low back pain" OR "neck pain" OR "shoulder pain"  #3 TI “placebo*” OR "open label placebo*" OR "open placebo*" OR "placebo response" OR "placebo effect*" OR "ethical use of placebo*" OR "no deceptive placebo*" OR "non decept" OR "nonconceal" OR "non conceal" OR "nonblind" OR "non blind" OR "without deception" OR "without conceal" OR "without blind" OR "sugar pill*" OR "placebo creme*" OR "placebo pill*"  #4 AB “placebo*” OR "open label placebo*" OR "open placebo*" OR "placebo response" OR "placebo effect*" OR "ethical use of placebo*" OR "no deceptive placebo*" OR "non decept" OR "nonconceal" OR "non conceal" OR "nonblind" OR "non blind" OR "without deception" OR "without conceal" OR "without blind" OR "sugar pill*" OR "placebo creme*" OR "placebo pill*" | 25/09/2023 ------------- 28/08/2024 | 960 --------- 11 |
| EMBASE | (#1) AND (#2) #1 TS= "chronic pain" OR "musculoskeletal pain" OR "long lasting pain" OR "intermittent pain" OR "long term pain" OR "persistent pain" OR "intractable pain" OR "nociceptive pain" OR "generalized pain" OR "widespread pain" OR "fibromyalgia" OR "myofascial pain syndrome" OR "myalgia" OR "idiopathic pain" OR "diffuse pain" OR “aspecific pain” OR "non specific pain" OR "nonspecific pain" OR "musculoskeletal pain syndrome" OR "chronic pain syndrome" OR "somatoform pain" OR "non cancer pain" OR "non malignant pain" OR "benign pain" OR "back pain" OR "low back pain" OR "neck pain" OR "shoulder pain"  #2 TS= "open label placebo*" OR "open placebo*" OR "placebo response" OR "ethical use of placebo*" OR "no deceptive placebo*" OR "non decept" OR "nonconceal" OR "non conceal" OR "nonblind" OR "non blind" OR "without deception" OR "without conceal" OR "without blind" OR "sugar pill*" OR "placebo creme*" OR "placebo pill*" | 25/09/2023 ------------- 28/08/2024 | 176 --------- 21 |
